# Human γδ T cells induce CD8^+^ T cell antitumor responses via antigen-presenting effect through HSP90-MyD88-mediated activation of JNK

**DOI:** 10.1007/s00262-023-03375-w

**Published:** 2023-01-21

**Authors:** Shengdong Wang, Hengyuan Li, Tao Chen, Hao Zhou, Wenkan Zhang, Nong Lin, Xiaohua Yu, Yu Lou, Binghao Li, Eloy Yinwang, Zenan Wang, Keyi Wang, Yucheng Xue, Hao Qu, Peng Lin, Hangxiang Sun, Wangsiyuan Teng, Haochen Mou, Xupeng Chai, Zhijian Cai, Zhaoming Ye

**Affiliations:** 1 Department of Orthopedics, Musculoskeletal Tumor Center, The Second Affiliated Hospital of Zhejiang University School of Medicine, Hangzhou, 310009 People’s Republic of China; 2 Institute of Orthopedic Research, Zhejiang University, Hangzhou, 310009 People’s Republic of China; 3 Key Laboratory of Motor System Disease Research and Precision Therapy of Zhejiang Province, Hangzhou, Zhejiang People’s Republic of China; 4 Department of Hepatobiliary and Pancreatic Surgery, The First Affiliated Hospital, Zhejiang University School of Medicine, Hangzhou, People’s Republic of China; 5 Institute of Immunology and Department of Orthopaedics of the Second Affiliated Hospital, Zhejiang University School of Medicine, Hangzhou, 310009 People’s Republic of China

**Keywords:** γδ T cells, Antigen presentation, Tumor immunotherapy, Osteosarcoma

## Abstract

Human Vγ9Vδ2 T cells have attracted considerable attention as novel alternative antigen-presenting cells (APCs) with the potential to replace dendritic cells in antitumor immunotherapy owing to their high proliferative capacity and low cost. However, the utility of γδ T cells as APCs to induce CD8^+^ T cell-mediated antitumor immune response, as well as the mechanism by which they perform APC functions, remains unexplored. In this study, we found that activated Vγ9Vδ2 T cells were capable of inducing robust CD8^+^ T cell responses in osteosarcoma cells. Activated γδ T cells also effectively suppressed osteosarcoma growth by priming CD8^+^ T cells in xenograft animal models. Mechanistically, we further revealed that activated γδ T cells exhibited increased HSP90 production, which fed back to upregulate MyD88, followed by JNK activation and a subsequent improvement in CCL5 secretion, leading to enhanced CD8^+^ T cell cross-priming. Thus, our study suggests that Vγ9Vδ2 T cells represent a promising alternative APC for the development of γδ T cell-based tumor immunotherapy.

**Supplementary Information:**

The online version contains supplementary material available at 10.1007/s00262-023-03375-w.

## Introduction

Cytotoxic CD8^+^ T cells are the most powerful effectors of the adaptive immune system, capable of destroying cancer cells in an antigen-specific fashion by interacting with major histocompatibility complex class-I (MHCI) molecules on the surface of antigen-presenting cells (APCs) and target cells. [1]. While direct presentation of tumor antigens on MHCI by tumor cells plays a pivotal role in effector function of CD8^+^ T cells, cross-presentation by professional APCs is required to prime naive CD8^+^ T cells and to sustain cytotoxic immune responses [2]. Because of their ability to initiate an immune response by cross-presenting tumor antigens, dendritic cells (DCs) are now considered as a core compartment of “cancer-immunity cycle” [3]. Despite the fact that DC-based therapy are being increasingly investigated in cancer clinical trials, limited success has been achieved due to its scarcity in peripheral blood, limited proliferative capacity and high costs related to DC induction and maturation[4]. Moreover, mounting evidence suggests that DCs are dysfunctional in the tumor microenvironment, which ultimately affects antitumor immune responses [5]. Therefore, novel alternative APCs are urgently required to improve the efficacy of cell-based immunotherapy for tumor.

Human Vγ9Vδ2 T cell is an important component of immune effector cells that contribute to immunosurveillance against tumors through direct recognition activity which relies on the engagement of TCR and/or natural killer cell receptors in an MHC-independent manner [6, 7]. Human γδ T cells, known for their potent innate effector functions, have been found to contribute to adaptive immunity by enhancing B-cell helper function, promoting DC maturation and inducing a rapid and transient expression of CCR7, the chemokine receptor enabling the interaction between naive/central memory T cells and mature DCs within the lymph nodes [8–11]. As a bridge between the innate and adaptive immune systems, human γδ T cell has been shown to display the characteristics of professional APCs. Brandes et al*.* reported for the first time that activated γδ T cells expressed phenotypic features of APCs, including adhesion receptors, co-stimulatory molecules and classic antigen-presenting molecules (MHC I and II) [12]. Brandes et al. and Meuter et al. further verified that these activated γδ T cells were highly effective in inducing CD4^+^ and CD8^+^ T cell responses, suggesting that activated human γδ T cells (γδ T-APCs) are a distinct type of APCs [13, 14]. Due to their relative abundance in peripheral blood and the availability of numerous approaches to selectively activate and reproduce human γδ T cells both in vitro and in vivo [15], γδ T-APCs hold great potential as a promising candidate for novel APCs in tumor immunotherapy.

Based on the APC-related characteristics of γδ T cells, researchers have begun to focus on the efficacy of these cells in inducing tumor-specific responses of CD8^+^ T cells through cross-presentation. Muto et al. reported that activated γδ T cells could induce antitumor CD8^+^ T cell responses using apoptotic tumor cells as the antigen resource [16]. Mao et al. further demonstrated that γδ T cells from gastric cancer patients displayed the APC-related features and functions [17]. Despite the fact that these findings are solely based on in vitro studies, they suggest the promise of γδ T-APCs in tumor immunotherapy. Nevertheless, in vivo proof-of-concept evidence of γδ T-APC-induced adaptive immunity against cancer is lacking. Thus, additional cytotoxicity experiments using different tumor cell lines and animal studies are required to confirm the antigen-presenting capacity of γδ T-APCs. In addition, the mechanism by which resting γδ T cells develop into APCs remains unclear.

Herein, we stimulated resting human γδ T cells with zoledronate (ZOL) and found that these activated γδ T cells markedly upregulated APC-related markers and cross-presented a tumor-derived peptide antigen. To clarify the efficacy of γδ T-APCs in inducing antitumor responses of CD8^+^ T cells, we used osteosarcoma cell lines as target cells and performed a lactate dehydrogenase (LDH) assay and 7-AAD staining to determine the cytotoxic activity of CD8^+^ T cells cross-primed by γδ T-APCs. Further, γδ T-APCs loaded with tumor lysates were used to induce the specific lysis of autologous CD8^+^ T cells against primary osteosarcoma cells from patients. In murine study, CD8^+^ T cells cross-primed by γδ T-APCs effectively suppressed osteosarcoma growth. The role of γδ T-APCs in adaptive antitumor immunity was clearly demonstrated in these cytotoxicity experiments. Mechanistically, our study is the first to reveal that ZOL-activated γδ T cells exhibit increased ﻿heat-shock protein 90 (HSP90) production, which provides feedback to upregulate MyD88, followed by JNK activation and CCL5 generation, thereby contributing to γδ T cell-mediated cross-presentation. Therefore, our findings shed light on the antigen-presenting potential of γδ T cells and suggest that as a novel APC, γδ T cells could be a promising alternative to DCs for the application in tumor immunotherapy.

## Materials and methods

### Mice

Immunodeficient NOD-SCID *IL2rg*^*null*^ (NSG) mice were purchased from the Shanghai Laboratory Animal Center of the Chinese Academy of Sciences. The mice were kept under specified pathogen-free conditions and supplied with sterilized food and water. Ethical approval was granted by the Institutional Animal Care and Use Committee, and the study was conducted in accordance with the ethical and legal rules.

### Cells and cell culture

This study was approved by the Human Research Ethics Committee of the Second Affiliated Hospital, School of Medicine, Zhejiang University (Hangzhou, China). This research was performed in accordance with the Declaration of Helsinki and the national and international guidelines. Written informed consent was obtained from all donors and osteosarcoma patients.

Peripheral blood mononuclear cells (PBMCs) were isolated from HLA-A0201-positive volunteers and osteosarcoma patients using density gradient centrifugation (Cedarlane Laboratories, Burlington, ON, Canada). According to our previous study [18], Vγ9Vδ2 T cells were stimulated with 1 µM zoledronate (Zometa®; Novartis International AG, Basel, Switzerland) in the presence of 400 IU/ml IL-2 (R&D, Minneapolis, USA) on the first day. The cultures were then replaced with fresh medium ﻿supplemented with IL-2 at the same concentration every three days. Following 7–10 days of culture, cells were harvested for antigen presentation assays. The TCRγ/δ^+^ T Cell Isolation Kit (Miltenyi Biotec GmbH, Bergisch Gladbach, Germany) was used to purify γδ T cells, and the purity of γδ T cells was determined by flow cytometry. DCs were generated from adherent cells cultured for 5 days in the presence of 1000 IU/ml GM-CSF and 10 ng/ml IL-4 (both R&D) and matured using a cocktail consisting of 10 ng/ml TNF-α, 10 ng/ml IL-1β, 10 ng/ml IL-6 (all R&D) and 1 μg/ml PGE-2 (Sigma-Aldrich, St. Louis, USA). All immune cells were maintained in RPMI 1640 medium (Gibco, Carlsbad, CA, USA) supplemented with 1% L-glutamine, 1% penicillin–streptomycin and 10% fetal bovine serum (Gibco).

The human osteosarcoma cell lines HOS, SAOS-2 and U2OS were obtained from the Cell Bank of the Shanghai Institute of Biochemistry and Cell Biology, Chinese Academy of Sciences (Shanghai, China). All cell lines were authenticated by short tandem repeat genotyping. HOS, SAOS-2 and primary osteosarcoma cells were cultured in DMEM (Gibco) supplemented with 1% L-glutamine, 1% penicillin–streptomycin and 10% fetal bovine serum, whereas the U2OS cells were maintained in RPMI 1640 medium. Cell lines were incubated at 37 °C in 5% CO_2_ and routinely tested for mycoplasma contamination using the Mycoplasma Detection Kit (Thermo Fisher Scientific, Waltham, MA, USA).

### Antigen presentation assay

Melanoma-associated antigen A3 (MAGEA3) peptide (FLWGPRALV, purity > 98%, Sangon Biotech, Shanghai, China) was used for the antigen presentation assay [19]. Purified naive CD8^+^ T cells were obtained from PBMCs of healthy donors for antigen presentation assays using the Miltenyi Naive CD8^+^ T Cell Isolation Kit. Briefly, γδ T-APCs or DCs were pretreated with or without 10 μg/ml MAGEA3 peptide for two hours, washed extensively and subsequently co-cultured with CD8^+^ T cells at an APC to responder cell ratio of 1:10 in the presence of 50 IU/ml IL-2 (R&D). Carboxyfluorescein diacetate succinimidyl ester (CFSE) (Thermo Fisher Scientific) labeling was performed to evaluate cell proliferation as described previously [13]. Briefly, CFSE-labeled CD8^+^ T cells were co-cultured with peptide non-pulsed APCs or MAGEA3-pulsed APCs for 10 days. The cells were washed and labeled with anti-CD8a mAb before flow cytometry. CD8^+^ T-cell proliferation was measured by detecting the CFSE^−^ CD8^+^ cell proportion. These experiments were reproduced 2–4 times using PBMCs derived from three individuals.

To detect the activation of CD8^+^ T cells, CD8^+^ T cells were co-cultured with APCs pretreated with or without peptides for 7 days. Then, the cells in each group received the same stimulation as the previous one for 4 h at 37 °C in the presence of 0.7 μl/ml GolgiStop containing Monensin (BD Biosciences). The cells were washed and labeled with anti-CD8a and anti-CD107a antibodies before being fixed and permeabilized using Cytofix/Cytoperm buffer (BD Pharmingen). After being washed twice in Perm/Wash buffer (BD Pharmingen), the cells were intracellularly stained for perforin or IFN-γ and detected by flow cytometry. To detect specific stimulation, CD8^+^ T cells isolated from PBMCs were co-cultured with peptide non-pulsed APCs, irrelevant peptide (IP)-pretreated APCs or MAGEA3-pretreated APCs for 10 days. MAGEA3-specific CD8^+^ T cell induction was evaluated by IFN-γ ELISpot analysis (R&D) and MHC I-restricted MAGEA3 tetramer staining following the manufacturer’s instructions. These experiments were reproduced 2–4 times using PBMCs derived from three individuals.

## Flow cytometry

Cells were harvested and washed with cell staining buffer (BioLegend, San Diego, CA, USA) before being incubated for 30 min in the dark at 4 °C with the indicated fluorochrome-conjugated antibodies. For intracellular staining, surface-stained cells were fixed with 4% paraformaldehyde and permeabilized with intracellular staining permeabilization wash buffer (BioLegend), and intracellular staining was carried out according to the manufacturer’s instructions. The cells were then washed twice with staining buffer at room temperature for 5 min and analyzed using flow cytometry (FACSCalibur, BD) with a minimum of 10,000 events collected. Flow cytometry data were analyzed using FlowJo V10.

Antibodies specific for the following proteins were used: TCR-Vδ2, HLA-ABC, HLA-DR, CD80, CD86, CD54, CD69, CD8, CD107a, perforin and IFN-γ. All conjugated antibodies were purchased from BioLegend. MAGEA3 tetramer was obtained from HelixGen (Guangzhou, China).

### Tumor lysates

Osteosarcoma cell pellets were resuspended in cold phosphate-buffered saline (PBS) at a density of 1 × 10^7^/mL and then subjected to five freeze–thaw cycles. The tumor lysates were centrifuged at 2000 g for 10 min to remove the debris. BCA assay (Thermo Fisher Scientific) was used to measure the protein concentration.

### Confocal microscopy

Γδ T-APCs were seeded into 96-well plates at a density of 1 × 10^5^ cells/well. The cells were pulsed with 10 μg/ml FITC-MAGEA3 (YEFLWGPRALVETSYVKVLHHM, purity > 98%) (Sangon Biotech) for 1 h and then washed twice with PBS before being cultured in fresh medium for an additional 1, 3, 6 or 24 h at 37 °C in 5% CO_2_. After being washed twice with PBS, cells were fixed with 4% paraformaldehyde for 20 min at room temperature, permeabilized and blocked for 30 min in 0.05% Triton X-100 and 2% bovine serum albumin. The cells were then washed and incubated with antibodies against specific markers for individual organelles, including anti-EEA1 to identify early endosomes, anti-Rab7 to identify late endosomes, anti-KDEL to identify the endoplasmic reticulum and anti-LAMP1 to identify lysosomes (all from Cell Signaling Technology, Beverly, MA, USA). The cells were then washed and incubated with a fluorochrome-conjugated secondary antibody (Cell Signaling Technology) for 2 h at room temperature. The samples were mounted with Prolong Gold antifade reagent with DAPI (Thermo Fisher Scientific) before being detected by confocal laser scanning microscopy (Olympus FV1200).

### RNA-seq analysis

RNA-seq was performed using Annoroad Genomics (Beijing, China). Briefly, RNA was extracted from purified resting γδ T cells and ZOL-activated γδ T cells using TRIzol reagent (Invitrogen) and was used for library preparation. Total RNA quality was determined by estimating the A260/A280 and A260/A230 ratios using NanoDrop (Thermo Scientific). The A260/A280 ratios were in the range of 1.9–2.0 and the A260/A230 ratios were higher than 2.0 for all samples. RNA-seq libraries were generated and indexed using the NEBNext Ultra RNA Library Prep Kit (NEB, USA) following the manufacturer’s recommendations. 150-bp paired-end sequencing was performed using a HiSeq2000 sequencer (Illumina). Sequencing data were aligned to the reference genome using HISAT2 (v2.1.0), followed by differentially expressed gene (DEG) identification using the Sleuth R package with a cutoff of *p* < 0.05. Relative gene expression level for the most abundant transcript of each gene is presented as transcripts per kilobase million (TPM). Gene ontology (GO) analysis (http://www.geneontology.org) was used to identify the biological functions of the DEGs. GO enrichment of DEGs was implemented using the hypergeometric test, in which the p value was calculated and adjusted as the q value. GO terms with *q* < 0.05 were considered to be significantly enriched. The Kyoto Encyclopedia of Genes and Genomes (KEGG, http://www.kegg.jp/) is a database resource that contains a collection of manually drawn pathway maps representing our knowledge of molecular interactions and reaction networks. KEGG enrichment of DEGs was implemented using the hypergeometric test, in which the p value was adjusted by multiple comparisons as the q value. KEGG terms with *q* < 0.05 were considered to be significantly enriched.

### RT-PCR

Total RNA was extracted from resting or ZOL-activated γδ T cells using TRIzol reagent following the manufacturer’s protocol. The RNA quality and concentration were measured using a NanoDrop 2000c spectrophotometer. cDNA was synthesized using the PrimeScript RT reagent kit (TaKaRa, China). Real-time PCR was performed using SYBR Green Master Mix (Roche, Switzerland) and a multicolor real-time PCR detection system (Bio-Rad, USA). Relative RNA levels were calculated using the 2^−ΔΔCT^ method and normalized to GAPDH expression. The primers used for RT-PCR are shown in Table S1 and were designed and synthesized by Sangon Biotech.

### Western blot

Γδ T cells with different treatments were centrifuged and lysed in RIPA buffer in the presence of a proteasome inhibitor. After quantification using the BCA assay, equal amounts of protein were separated by SDS-PAGE and transferred to polyvinylidene difluoride membranes (Millipore Sigma, MA, USA). After being blocked with 5% bovine serum albumin in Tris-buffered saline with Tween 20 (TBST) for 2 h, the membranes were incubated with primary antibodies at 4 °C overnight. The membranes were washed with TBST and incubated with HRP-conjugated secondary antibodies at room temperature for 1 h. The targeted bands were visualized using an enhanced chemiluminescence detection system (ChemiDoc™ XRS + imaging system; Bio-Rad).

The primary antibodies against human HSP90α, HSP90β, GRP94, TRAP1 and MyD88 were purchased from Abcam. Primary antibodies against human JNK, phospho-JNK, ERK, phospho-ERK, P38, phospho-P38 and GAPDH were purchased from Cell Signaling Technology. All primary antibodies were diluted to 1:1000.

### Cell transfection

Osteosarcoma cell lines were transfected with MAGEA3 for the cytotoxicity assays. For lentivirus-mediated overexpression of MAGEA3 in osteosarcoma cells, full-length human MAGEA3 (NM_005362.3) was inserted into the pHBLV-CMV-MCS-3FLAG-EF1-ZsGreen-T2A-PURO lentiviral vector (Hanbio Biotechnology, Shanghai, China). For the in vivo study, HOS cells were co-transfected with luciferase. Briefly, osteosarcoma cells were plated at a density of 4 × 10^5^ cells/well in a 6-well plate 18 h before transfection. The cells were transfected with recombinant lentiviruses at a multiplicity of infection (MOI) of 10:1 in the presence of 8 μg/ml polybrene according to the manufacturer's instructions. The cells were treated with puromycin (1 μg/ml) for two days for selection, which eliminated all the cells in the uninfected control group. Protein samples were collected for western blot analysis to detect MAGEA3 expression. An in vivo imaging system (Lumina Series III, Caliper Life Sciences) was used to measure the bioluminescence of the luciferase-transfected cells.

### Cytotoxicity assay

The cytotoxicity of CD8^+^ T cells against osteosarcoma cells was measured by LDH release assay (Beyotime, China) according to the manufacturer’s instructions. Briefly, CD8^+^ T cells isolated from PBMCs were, respectively, co-cultured with γδ T-APCs, irrelevant peptide-pretreated γδ T-APCs (γδ T-IP), MAGEA3-pretreated γδ T-APCs (γδ T-MAGEA3) or MAGEA3-pretreated DCs (DC-MAGEA3) in the presence of IL-2. After 7 days of co-culture, the cells in each group were, respectively, treated with γδ T-APCs, γδ T-IP, γδ T-MAGEA3 or DC-MAGEA3 again with the addition of IL-2 for another 7 days. The cells were then assessed for cytotoxicity. Osteosarcoma cells (3 × 10^3^ cells/well) were seeded in 96-well plates as target cells for CD8^+^ T cells at an effector/target ratio of 10:1. After 4 h of co-culture, LDH release was then determined using an LDH cytotoxicity assay kit and the percentage of specific lysis was calculated as follows: [(experimental release-spontaneous release) / (maximum release- spontaneous release)] × 100%. In addition, a 7-AAD/CFSE cytotoxicity assay was used to assess the cytolytic activity of T cells. Osteosarcoma cells (5 × 10^4^ cells/well) were seeded in 24-well plates and labeled with CFSE before being co-cultured with T cells. After 4 h of co-culture, the cells were incubated with a 7-AAD (BD Biosciences) for 20 min at 4 °C in the dark, and the lysis of CFSE-positive tumor cells was evaluated by flow cytometry (FACSCalibur, BD).

## In vivo study

Healthy 4-week-old female NSG mice were injected subcutaneously with 5 × 10^6^ HOS cells co-transfected with MAGEA3 and luciferase. The mice were randomly separated into three groups (five mice per group). On the 7th day after tumor cell injection, the mice in each group began to receive the following treatments: [1] PBS (untreated mice), [2] 5 × 10^7^ CD8^+^ T cells that had been co-cultured with peptide non-pulsed γδ T-APCs, [3] 5 × 10^7^ CD8^+^ T cells that had been co-cultured with γδ T-IP and [4] 5 × 10^7^ CD8^+^ T cells that had been co-cultured with γδ T-MAGEA3. For in vivo tracking, human CD8^+^ T cells were labeled with XenoLight DiR (Caliper Life Sciences, Hopkinton, USA), and then, these DiR-labeled cells were adoptively transferred into tumor-bearing mice via intravenous injection. PBS or CD8^+^ T cells were administered via the tail vein every two days for a total of three infusions. Tumors were measured with a caliper every two days, and tumor volume was estimated using the following formula: volume = (length × width^2^)/2. On the 10th, 13th and 16th days, the mice in each group were imaged using an in vivo imaging system to detect the bioluminescence of luciferase-transfected tumor tissue and the fluorescence of DiR-labeled T cells. On the 18^th^ day, all mice were killed, and the tumors were excised and fixed in formalin for further analysis.

### Immunohistochemical analysis

Formalin-fixed, paraffin-embedded tumor specimens were cut into serial sections with a thickness of 3 µm. IHC staining with anti-CD8 antibody (Abcam, MA, USA) at a dilution of 1:50 was performed on consecutive tissue sections to visualize intratumoral CD8^+^ T cells. Images were captured using a microscope. The quantitation of the positive cells in the tumor sections was performed by counting the number of positive cells in at least five fields of view at a high magnification (40 × objective lens), and the average number of positive cells per field was calculated.

### Statistical analyses

For relevant pairwise comparisons, paired or unpaired Student’s t test was performed. One-way analysis of variance (ANOVA) was performed to compare multiple groups. Tetramer staining and tumor growth curves were compared using two-way ANOVA. *p* < 0.05 was considered statistically significant. All values are presented as mean ± SD. All the statistical analyses were conducted via GraphPad Prism 8 and SPSS 21.0.

## Results

### Activated γδ T cells show antigen-presenting potential.

Purified γδ T cells (purity of Vδ2 T cells > 95%) were obtained from PBMCs using magnetic-activated cell sorting (Figure S1A), termed resting γδ T cells. We first profiled transcriptional changes using RNA-seq analysis and showed that 7422 genes displayed differential expression patterns in ZOL-stimulated γδ T cells compared with resting γδ T cells (Figure S1B). GO analysis of DEGs revealed a close association between antigen processing and presentation (Figure S1C). To confirm whether activated γδ T cells display the features of professional APCs, we stimulated γδ T cells with ZOL and observed signs of preactivation (surface CD69 expression) and upregulation of APC-related markers, including antigen-presenting MHC molecules (HLA-ABC and HLA-DR), co-stimulatory molecules (CD80 and CD86) and adhesion molecules (CD54) (Figure S2A-C). Furthermore, immunocytochemical analysis of resting and activated γδ T cells revealed increased synthesis of HLA-ABC (Figure S2D), which is pivotal for inducing CD8^+^ T cell responses. In addition, our results revealed that γδ T cells displayed a much greater proliferative capacity than DCs (Figure S2E). Consistent with previous studies, these results suggest that γδ T cells have the potential to perform antigen presentation and undergo rapid expansion.

### γδ T-APCs effectively cross-prime CD8^+^ T cells

**Fig. 1:**
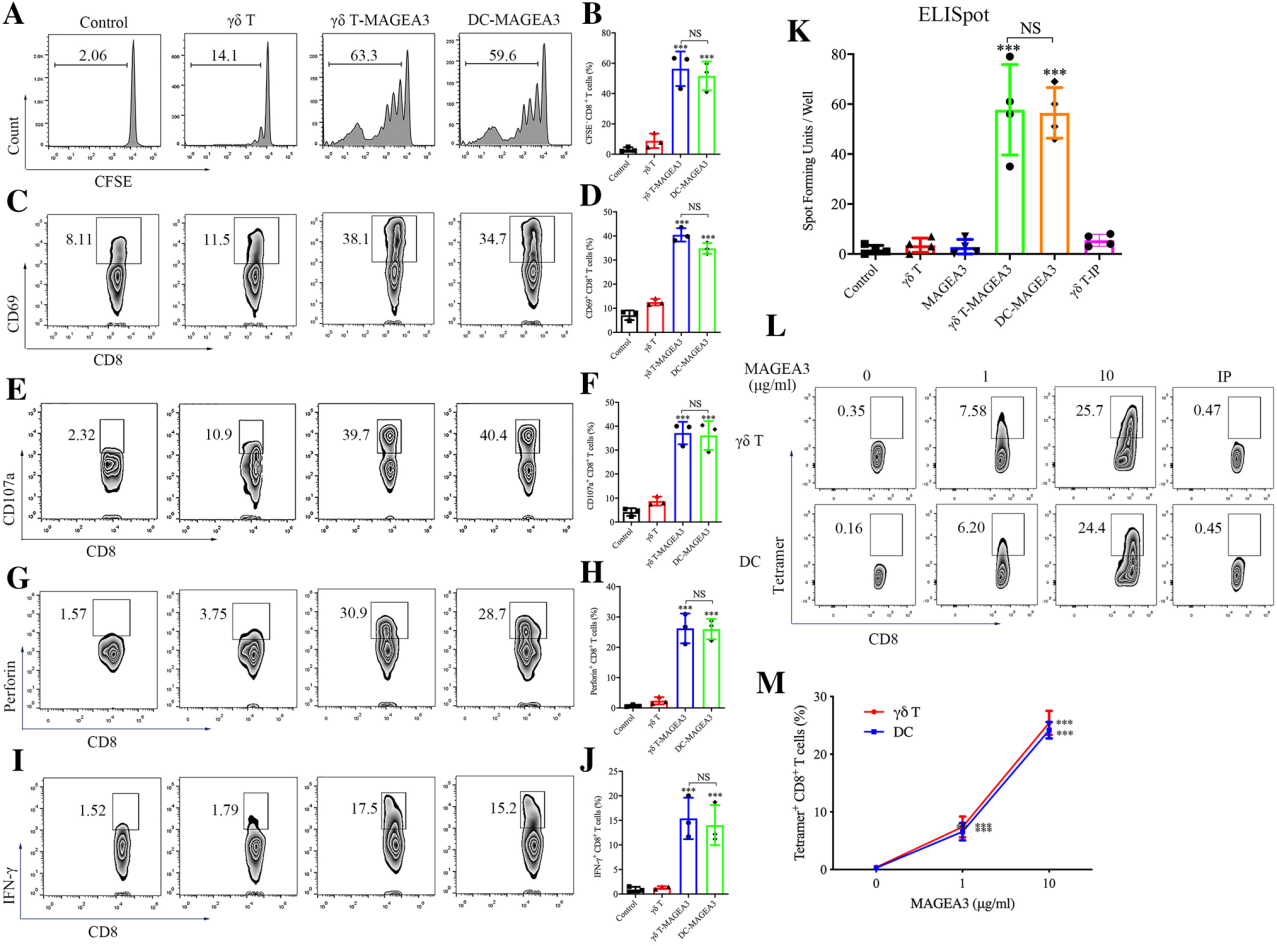
γδ T-APCs induce CD8^+^ T cell proliferation and activation. γδ T cells stimulated by zoledronate and IL-2 for 10 days were used as γδ T-APCs. **A**, **B** γδ T-APCs or DCs were, respectively, pretreated for 2 h with 10 μg/ml MAGEA3 peptide (γδ T-MAGEA3 and DC-MAGEA3) and then washed extensively. After that, peptide non-pulsed γδ T-APCs (γδ T), γδ T-MAGEA3 and DC-MAGEA3 were then co-cultured with CFSE-labeled CD8^+^ T cells for 10 days at an APC/responder cell ratio of 1:10. Proliferating CFSE^−^ CD8^+^ T cells were measured by flow cytometry. **C**, **D** γδ T-APCs or DCs were pretreated for 2 h with 10 μg/ml MAGEA3 peptide and then washed extensively. After that, peptide non-pulsed γδ T-APCs, γδ T-MAGEA3 and DC-MAGEA3 were then co-cultured with CD8^+^ T cells for 5 days at an APC/responder cell ratio of 1:10. CD69 expression levels on CD8^+^ T cells were measured by flow cytometry. **E–J** γδ T-APCs or DCs were pretreated for 2 h with 10 μg/ml MAGEA3 peptide and then washed extensively. After that, peptide non-pulsed γδ T-APCs, γδ T-MAGEA3 and DC-MAGEA3 were then co-cultured with CD8^+^ T cells for 7 days at an APC/responder cell ratio of 1:10. After that, the cells in each group received the same stimulation as the previous one for 4 h followed by further analysis. **E**, **F** CD107a expression levels on CD8^+^ T cells were measured by flow cytometry. **G**, **H** Perforin expression levels in CD8^+^ T cells were measured by flow cytometry. **I**, **J** IFN-γ expression levels in CD8^+^ T cells were measured by flow cytometry. **K** CD8^+^ T cells were treated with MAGEA3 peptide or co-cultured, respectively, with peptide non-pulsed γδ T-APCs, irrelevant peptide-pulsed γδ T-APCs (γδ T-IP), γδ T-MAGEA3 or DC-MAGEA3 for 10 days at an APC/responder cell ratio of 1:10. Specificity of the generated MAGEA3-specific T cells was assessed by IFN-γ ELISpot. Cells were plated in triplicates at 10,000 cells per well of anti-IFN-γ capture antibody-coated plates and IFN-γ spot-forming units/well was shown in histogram. **L**, **M** γδ T-APCs or DCs were pretreated with indicated concentrations of MAGEA3 peptide or 10 μg/ml irrelevant peptide (IP) for 2 h, then washed extensively and co-cultured with CD8^+^ T cells for 10 days at an APC/responder cell ratio of 1:10. MAGEA3-specific responder cells were quantified by MAGEA3-tetramer staining using flow cytometry. Representative data were shown from 2 to 4 independent experiments. All the values were presented as mean ± SD. ***p < 0.001 versus Control or 0 μg/ml of the corresponding group (M). NS, not significant

Next, we evaluated intracellular antigen trafficking in γδ T cells using confocal microscopy. As shown in Figure S3, FITC-conjugated long peptides co-localized with early endosomes (EEA1^+^), late endosomes (Rab7^+^), endoplasmic reticulum (KDEL^+^) and lysosomes (Lamp1^+^) after incubation with γδ T cells, which was similar to the antigen peptide localization in classic APCs [20, 21]. However, the ability of γδ T-APCs to induce antitumor responses of CD8^+^ T cells remains unknown. To investigate this, we studied the ability of γδ T-APCs to cross-present MAGEA3, a cancer–testis antigen that has been widely used in clinical trials for tumor vaccines [22]. Specifically, we used DCs as the positive control. Remarkably, CD8^+^ T cells showed marked proliferation after co-culture with γδ T-MAGEA3 or DC-MAGEA3, as assessed by monitoring the dilution of CFSE (Fig. 1A, B). In addition to expansion, CD8^+^ T cells cross-primed by γδ T-MAGEA3 also upregulated the early activation marker CD69 (Fig. 1C, D) and the degranulation marker CD107a (Fig. 1E, F). Moreover, we found that γδ T-MAGEA3 induced robust perforin and IFN-γ production in CD8^+^ T cells, indicating the activation of functional CD8^+^ T cells (Fig. 1G–J). Correspondingly, we performed an ELISpot assay to assess the specificity of stimulated T cells and found that γδ T-MAGEA3 greatly increased the number of spot-forming units per well (Fig. 1K). Moreover, we observed a significant increase in MAGEA3-specific CD8^+^ T cells after γδ T-MAGEA3 priming using tetramer staining (Fig. 1L, M). Importantly, γδ T-APCs were shown to possess the capability to cross-prime CD8^+^ T cells comparable to that of DCs.

### γδ T-APCs induce the cytotoxic activity of CD8^+^ T cells against osteosarcoma cells

**Fig. 2:**
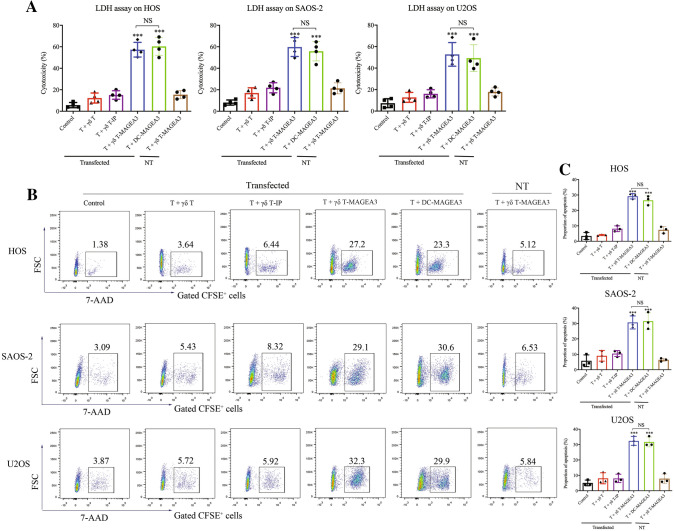
γδ T-APCs induce the cytotoxicity of CD8^+^ T cells against osteosarcoma cells. γδ T cells stimulated by zoledronate and IL-2 for 10 days were used as γδ T-APCs. γδ T-APCs or DCs were, respectively, pretreated for 2 h with 10 μg/ml MAGEA3 peptide (γδ T-MAGEA3 and DC-MAGEA3) or irrelevant peptide (γδ T-IP) and then washed extensively before co-culture. CD8^+^ T cells isolated from PBMCs were, respectively, co-cultured with peptide non-pulsed γδ T cells, γδ T-IP, γδ T-MAGEA3 or DC-MAGEA3 for 14 days at an APC/responder cell ratio of 1:10. Then, T cells were harvested and co-cultured with MAGE-A3-transfected or non-transfected (NT) osteosarcoma cell lines for 4 h at an effector/target cell ratio of 10:1. **A** The antitumor effect was measured by LDH assay. **B**, **C** Osteosarcoma cells were labeled with CFSE and then incubated with T cells for 4 h at an effector/target cell ratio of 10:1. Then, the cells after co-culture were stained with 7-AAD and the lysis of CFSE-positive tumor cells was evaluated using flow cytometry. Representative data were shown from 3 independent experiments. All the values were presented as mean ± SD. ***p < 0.001 versus Control. NS, not significant

It is well known that osteosarcoma induces poor antitumor immunity. To more convincingly verify the antigen-presenting capacity of γδ T-APCs, we tested whether γδ T-APCs could induce antitumor responses of CD8^+^ T cells against osteosarcoma cells. Since osteosarcoma-specific antigens remain unknown, MAGEA3 was ectopically expressed in osteosarcoma cell lines. CD8^+^ T cells were, respectively, co-cultured with γδ T-APCs, γδ T-IP, γδ T-MAGEA3 or DC-MAGEA3 before being co-cultured with MAGEA3-transfected or non-transfected osteosarcoma cells. As shown in Fig. 2A, CD8^+^ T cells cross-primed by γδ T-MAGEA3 or DC-MAGEA3 showed robust but comparable cytotoxicity against diverse MAGEA3^+^ osteosarcoma cell lines when evaluated by the LDH release assay. The cytotoxicity of CD8^+^ T cells was further determined using the 7-AAD/CFSE assay to evaluate tumor cell death (Fig. 2B, C). Similar antitumor effect was shown when using HER2 as another epitope (Figure S4A). Therefore, these results demonstrate the remarkable capacity of γδ T-APCs to induce antitumor immunity.

Next, we detected the effect of γδ T-APCs in inducing the cytotoxicity of CD8^+^ T cells against primary osteosarcoma cells. Lysates of tumor tissues from osteosarcoma patients were used for in vitro priming. The information of osteosarcoma patients is shown in Table S2 in Supplementary Material. We first showed that CD8^+^ T cells cross-primed by lysate-pulsed γδ T cells (γδ T-Lysate) exerted a significant cytotoxic effect against osteosarcoma cell lines. Then similar effect was found in autologous T cells against primary tumor cells from osteosarcoma patients (Figure S4B). These results suggest the potential of clinical application of γδ T-APCs for osteosarcoma immunotherapy.

### γδ T-APCs induce CD8^+^ T cell-mediated antitumor effect against osteosarcoma in vivo

**Fig. 3:**
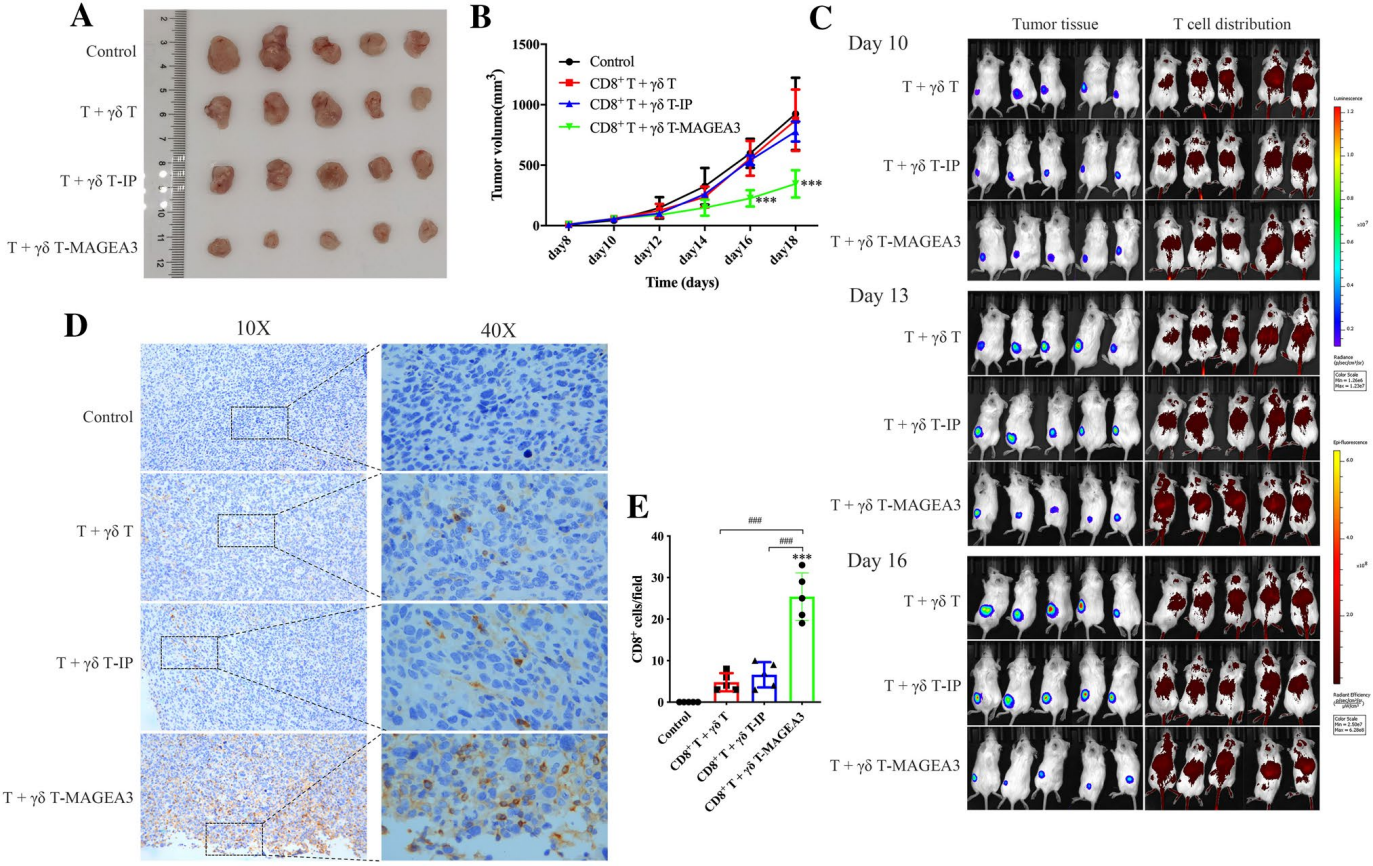
γδ T-APCs induce CD8^+^ T cell-mediated antitumor effects against osteosarcoma in vivo. HOS cells co-transfected with MAGEA3 and luciferase were inoculated subcutaneously into the left thighs of NOD-SCID mice. γδ T cells stimulated by zoledronate and IL-2 for 10 days were used as γδ T-APCs. After 7 days, mice started to receive the injection of CD8^+^ T cells, respectively, stimulated by peptide non-pulsed γδ T-APCs, irrelevant peptide-pretreated γδ T-APCs (γδ T-IP) or MAGEA3-pretreated γδ T-APCs (γδ T-MAGEA3) via the tail vein. **A** Mice were euthanized on the 18th day and the tumors were excised. **B** Tumor volumes were measured every 2 days, starting on the 8th day. **C** Mice were imaged with the in vivo imaging system on the 10th, 13th and 16th days. Tumor growth was evaluated by visualizing bioluminescence, and T cell migration was detected by DiR fluorescence. **D** Intratumoral CD8^+^ T cells were detected by immunohistochemical assays (shown in brown). **E** Quantification of CD8^+^ T cells infiltration was shown in histogram. Representative data were shown from 2 independent experiments. All the values were presented as mean ± SD. ***p < 0.001 versus Control. ###*p* < 0.001

To evaluate the capacity of γδ T-APCs to induce CD8^+^ T cell-mediated cytotoxicity in osteosarcoma cells in vivo, HOS cells transfected with MAGEA3 and luciferase (Figure S5A, B) were used to establish tumor-bearing mouse models. As shown in Fig. 3A, B, CD8^+^ T cells primed by γδ T-MAGEA3 significantly inhibited osteosarcoma growth. Furthermore, the in vivo imaging system showed increased T cell homing to the tumor site and better tumor control in mice treated with γδ T-MAGEA3-primed CD8^+^ T cells (Fig. 3C). In addition, the upregulated levels of infiltrating CD8^+^ T cells in tumor tissues from mice treated with γδ T-MAGEA3-primed CD8^+^ T cells were further confirmed by IHC (Fig. 3D, E). These results suggest that γδ T-APCs could effectively prime tumor-specific CD8^+^ T cells, which could be used to treat osteosarcoma as adoptive cell therapy.

### ZOL-stimulated γδ T cells obtain APC functions in an HSP90-dependent manner

**Fig. 4:**
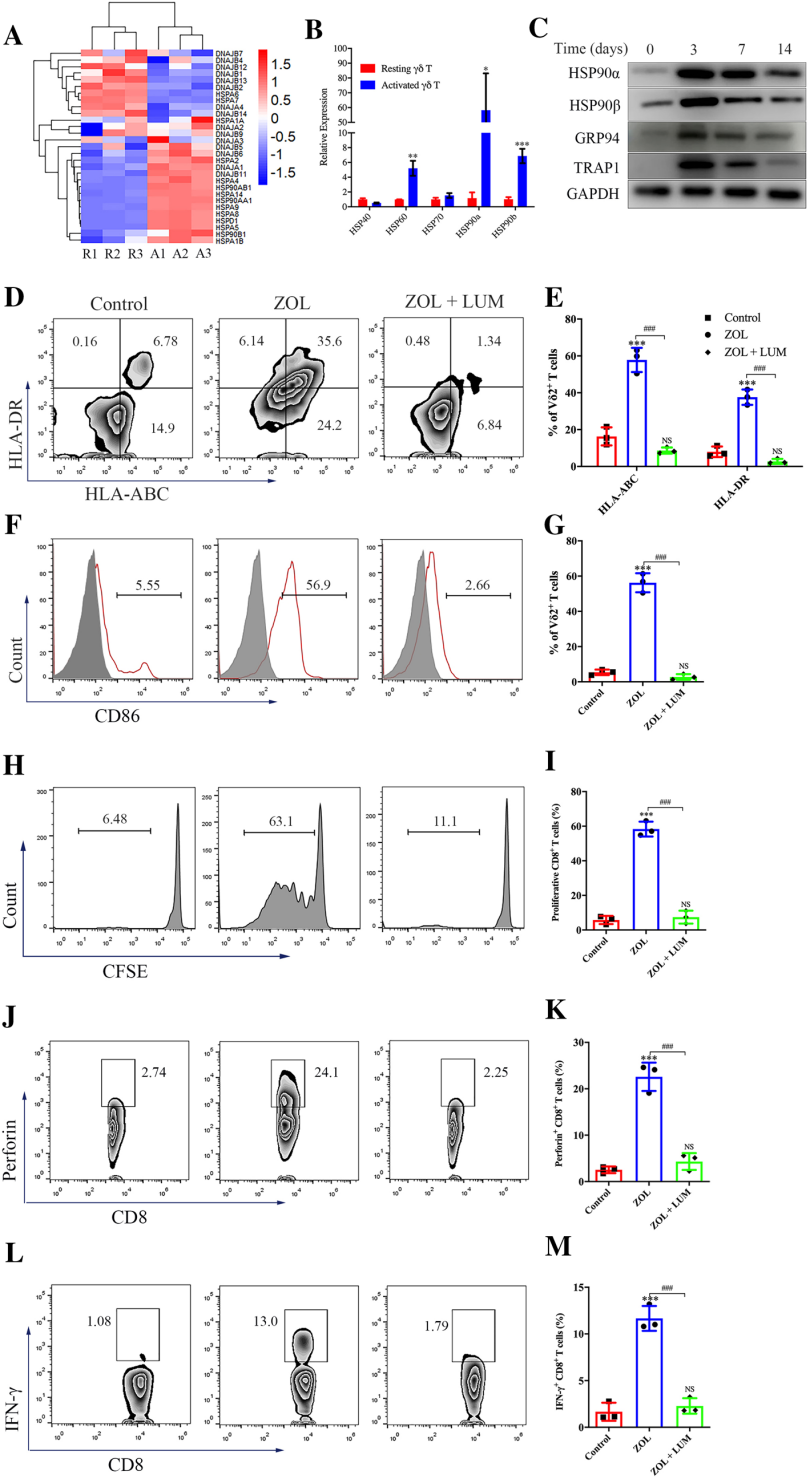
Zoledronate (ZOL)-stimulated γδ T cells obtain APC functions in an HSP90-dependent manner. **A** RNA extracted from purified resting γδ T cells and ZOL-activated γδ T cells was obtained for sequencing. Heatmap of gene expression profile by RNA sequencing showed the differential expression of *HSP* genes in resting γδ T cells (R1-3) and ZOL-stimulated γδ T cells (A1-3). **B** RT-PCR showed the expression levels of *HSP* genes in resting γδ T cells and ZOL-stimulated γδ T cells. **C** Resting γδ T cells were treated with 1 μM ZOL for indicated days. The expression levels of HSP90α, HSP90β, GRP94 and TRAP1 were detected by western blot. **D**–**G** Resting γδ T cells were treated with ZOL or ZOL plus HSP90 inhibitor Luminespib (ZOL + LUM) for 3 days. **D**, **E** The expression levels of HLA molecules on γδ T cells were measured using flow cytometry. **F**, **G** The expression levels of CD86 on γδ T cells were measured using flow cytometry. **H**, **I** Resting γδ T cells were treated with ZOL or ZOL + LUM for 7 days before MAGEA3 incubation. Then, the γδ T cells were washed and co-cultured with CFSE-labeled CD8^+^ T cells for 10 days at an APC/responder cell ratio of 1:10. Proliferating CFSE^−^ CD8^+^ T cells were measured by flow cytometry. **J**–**M** Resting γδ T cells were treated with ZOL or ZOL + LUM for 7 days before MAGEA3 incubation. Then, the γδ T cells were washed and co-cultured with CD8^+^ T cells for 7 days at an APC/responder cell ratio of 1:10. After that, the cells in each group received the same stimulation as the previous one for 4 h followed by further analysis. **J**, **K** Perforin expression levels in CD8^+^ T cells were measured by flow cytometry. **L**, **M** IFN-γ expression levels in CD8^+^ T cells were measured by flow cytometry. Representative data were shown from 2 to 3 independent experiments. All the values were presented as mean ± SD. *p < 0.05, **p < 0.01, ***p < 0.001 versus Resting γδ T or Control, ###*p* < 0.001. NS, not significant versus Control

Heat-shock proteins (HSPs) are critical for antigen processing and presentation [23, 24]. After examining the HSP profile at the mRNA level using RNA-seq, we found that *HSP90* family genes were markedly upregulated in activated γδ T cells (Fig. 4A). HSP90 and its homologues have been shown to play pivotal roles in the regulation of surface APC phenotypic markers, cytosolic translocation and cross-presentation of exogenous antigens [24–26]. First, we confirmed that the levels of HSP90s in untreated resting γδ T cells were very low (Figure S8A) but notably increased after ZOL stimulation (Fig. 4B, C). We then investigated the role of HSP90 in inducing the antigen-presenting potential of γδ T cells. No significant difference was observed in the proliferation or apoptosis of γδ T cells in the presence of 50 nM luminespib (LUM), an HSP90 inhibitor (Figure S6A, B). However, after treatment with LUM, ZOL-induced upregulation of MHC molecules and CD86 in γδ T cells was significantly reduced (Fig. 4D–G). Furthermore, the ZOL-induced effect of γδ T-APCs to drive the proliferation and perforin and IFN-γ production of CD8^+^ T cells was completely abolished by LUM (Fig. 4H–M). Taken together, these results suggest that ZOL-activated γδ T cells promote the maturation of their APC functions via self-feedback mediated by HSP90.

### HSP90 mediates APC function development in ZOL-stimulated γδ T cells via the MyD88 pathway

Toll-like receptors (TLRs), especially TLR4, are one of the main families of receptors known to be connected to HSP90 [27, 28]. Additionally, previous studies have highlighted the involvement of TLR signaling in γδ T cell functions [29]. To investigate whether TLR4 is involved in γδ T cell-mediated antigen presentation, we treated resting γδ T cells with the TLR4 agonist LPS and found that LPS markedly increased the expression of MHC molecules and CD86 on γδ T cells (Figure S7). We further verified that TRIF was not involved in the induction of antigen presentation by γδ T cells using the TLR3 agonist PIC (Figure S7). Thus, ZOL-activated γδ T cells probably perform antigen-presenting functions through the MyD88 pathway.

**Fig. 5:**
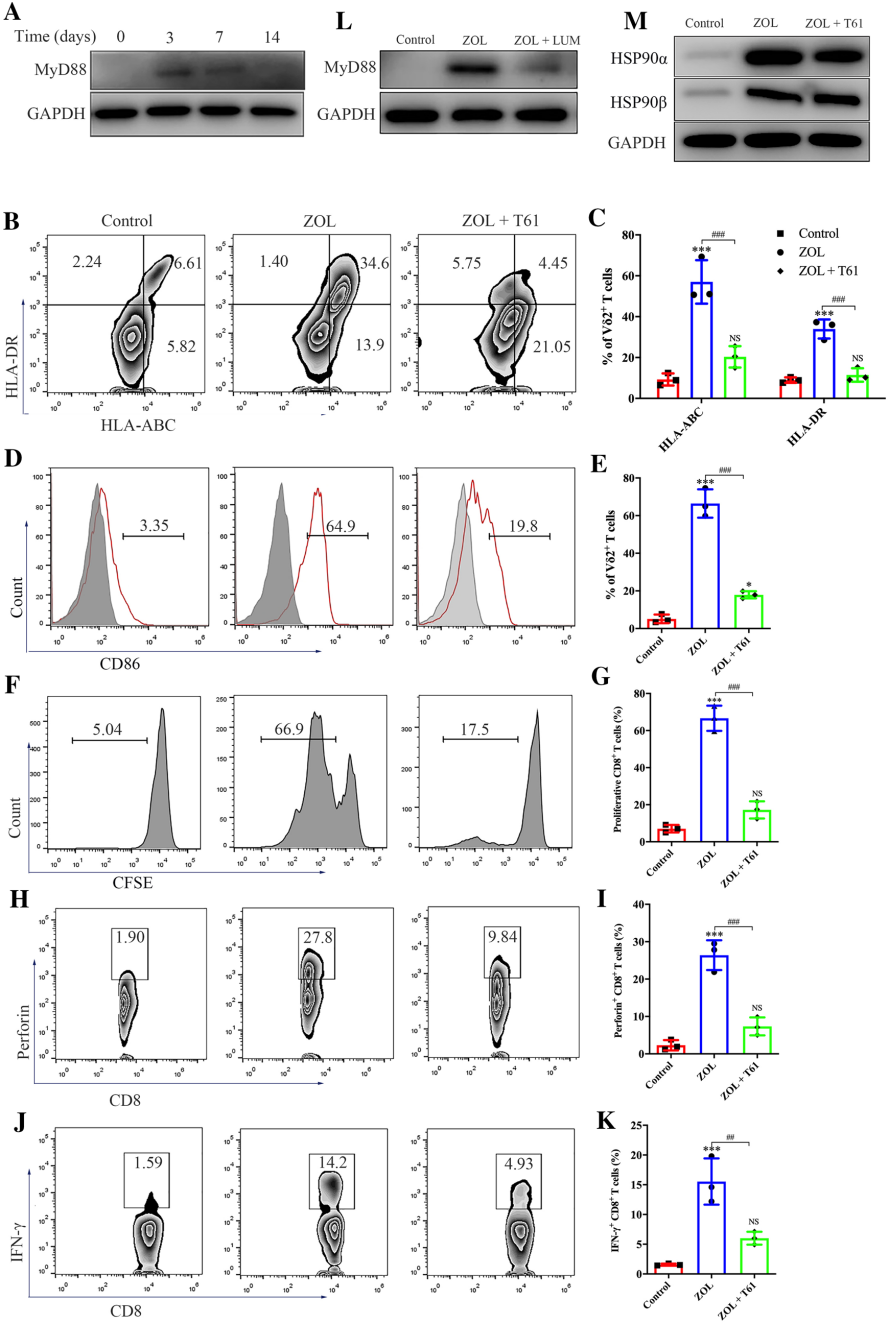
Zoledronate (ZOL)-stimulated γδ T cells obtain APC functions via the HSP90-MyD88 pathway. **A** Resting γδ T cells were treated with 1 μM ZOL for indicated days. The expression level of MyD88 was detected by western blot. **B**–**E** Resting γδ T cells were treated with ZOL or ZOL plus MyD88 inhibitor T6167923 (ZOL + T61) for 3 days. **B**, **C** The expression levels of MHC molecules on γδ T cells were measured using flow cytometry. **D**, **E** The expression levels of CD86 on γδ T cells were measured using flow cytometry. **F**, **G** Resting γδ T cells were treated with ZOL or ZOL + T61 for 7 days before MAGEA3 incubation. Then, the γδ T cells were washed and co-cultured with CFSE-labeled CD8^+^ T cells for 10 days at an APC/responder cell ratio of 1:10. Proliferating CFSE^−^ CD8^+^ T cells were measured by flow cytometry. **H**–**K** Resting γδ T cells were treated with ZOL or ZOL + T61 for 7 days before MAGEA3 incubation. Then, the γδ T cells were washed and co-cultured with pan T cells for 7 days at an APC/responder cell ratio of 1:10. After that, the cells in each group received the same stimulation as the previous one for 4 h followed by further analysis. **H**, **I** Perforin expression levels in CD8^+^ T cells were measured by flow cytometry. **J**, **K** IFN-γ expression levels in CD8^+^ T cells were measured by flow cytometry. **L** Resting γδ T cells were treated, respectively, with ZOL or ZOL + LUM for 7 days. The expression level of MyD88 was detected by western blot. **M** Resting γδ T cells were treated, respectively, with ZOL or ZOL + T61 for 7 days. The expression levels of HSP90α and HSP90β were detected by western blot. Representative data were shown from 2 to 3 independent experiments. All the values were presented as mean ± SD. *p < 0.05, ***p < 0.001 versus Control, ##*p* < 0.01, ###*p* < 0.001. NS, not significant vs. Control

We confirmed that ZOL significantly enhanced MyD88 protein levels in γδ T cells (Fig. 5A). We also found that the MyD88 inhibitor T6167923 (T61) markedly inhibited ZOL-induced upregulation of MHC molecules and CD86 on γδ T cells (Fig. 5B–E). Moreover, in the presence of T61, ZOL-stimulated γδ T cells showed a reduced ability to promote the proliferation and perforin and IFN-γ production of CD8^+^ T cells (Fig. 5F–K). MyD88 was further demonstrated to act as the downstream effector of HSP90; specifically, LUM significantly decreased MyD88 protein expression, whereas T61 had little effect in reducing HSP90 protein expression in ZOL-activated γδ T cells (Fig. 5L and M). Taken together, these findings demonstrate the key role of the HSP90-MyD88 pathway in the antigen-presenting activity of γδ T cells.

### ZOL endows γδ T cells with antigen-presenting activity via MyD88-mediated JNK activation.

**Fig. 6:**
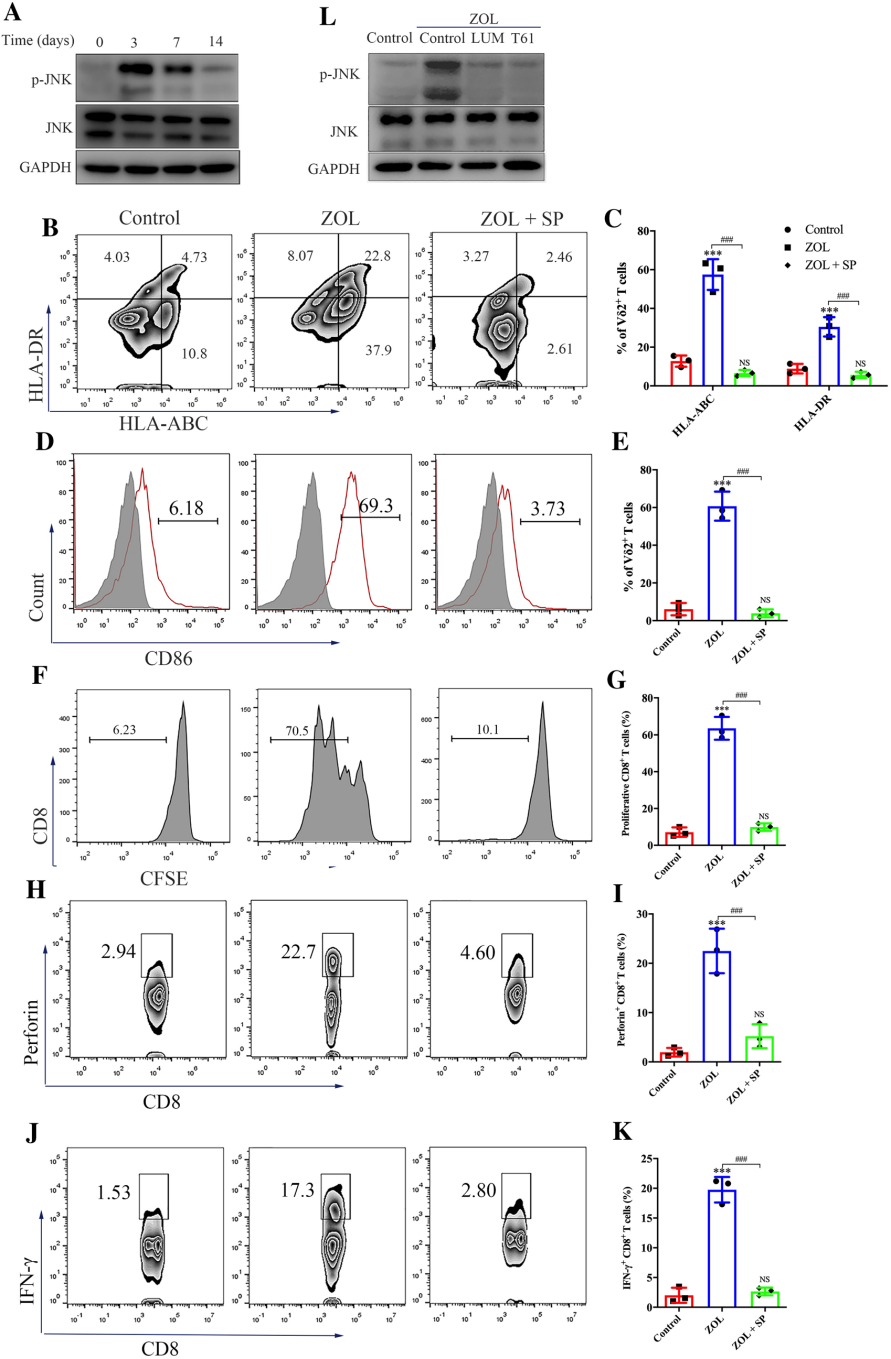
Zoledronate (ZOL) induces the antigen-presenting effect of γδ T cells via MyD88-mediated JNK activation. **A** Resting γδ T cells were treated with 1 μM ZOL for indicated days. The expression levels of phospho-JNK and total JNK were detected by western blot. **B**–**E** Resting γδ T cells were treated with ZOL or ZOL plus JNK inhibitor SP600125 (ZOL + SP) for 3 days. **B**, **C** The expression levels of MHC molecules on γδ T cells were measured using flow cytometry. **D**, **E** The expression levels of CD86 on γδ T cells were measured using flow cytometry. **F**, **G** Resting γδ T cells were treated with ZOL or ZOL + SP for 7 days before MAGEA3 incubation. Then, the γδ T cells were washed and co-cultured with CFSE-labeled CD8^+^ T cells for 10 days at an APC/responder cell ratio of 1:10. Proliferating CFSE^−^ CD8^+^ T cells were measured by flow cytometry. (H–K) Resting γδ T cells were treated with ZOL or ZOL + SP for 7 days before MAGEA3 incubation. Then, the γδ T cells were washed and co-cultured with CD8^+^ T cells for 7 days at an APC/responder cell ratio of 1:10. After that, the cells in each group received the same stimulation as the previous one for 4 h followed by further analysis. **H**, **I** Perforin expression levels in CD8^+^ T cells were measured by flow cytometry. **J**, **K** IFN-γ expression levels in CD8^+^ T cells were measured by flow cytometry. **L** Resting γδ T cells were treated, respectively, with ZOL, ZOL + LUM or ZOL + T61 for 7 days. The expression levels of p-JNK and JNK were detected by western blot. All the values were presented as mean ± SD. ***p < 0.001 versus Control, ###*p* < 0.001. NS, not significant versus Control

To further explore the mechanism of γδ T cell-mediated antigen presentation, we performed KEGG pathway enrichment analysis of RNA-seq data. As shown in Figure S8B, DEGs were enriched in several important classic signaling pathways, including the MAPK pathway. Hence, we investigated the alterations in the activities of P38, ERK and JNK in ZOL-treated γδ T cells. A slight upregulation of the phosphorylated P38 level and no change in the phosphorylated ERK level were observed in ZOL-stimulated γδ T cells (Figure S8C). Neither the P38- nor ERK-specific inhibitor has affected the expression of MHC molecules or CD86 on ZOL-stimulated γδ T cells (Figure S8D–G). In sharp contrast, the phosphorylated JNK level significantly increased in ZOL-stimulated γδ T cells (Fig. 6A). Furthermore, the JNK-specific inhibitor SP600125 completely attenuated the ZOL-induced upregulation of MHC molecules and CD86 on γδ T cells (Fig. 6B–E), and activated γδ T cells failed to induce CD8^+^ T cell proliferation and perforin and IFN-γ production in this context (Fig. 6F–K). Finally, both LUM and T61 effectively blocked JNK activation in ZOL-stimulated γδ T cells (Fig. 6L). Altogether, these results indicate that the antigen-presenting function of ZOL-activated γδ T cells is regulated by JNK activation via the HSP90-MyD88 axis.

### CCL5 promotes the antigen-presenting function of γδ T cells

**Fig. 7:**
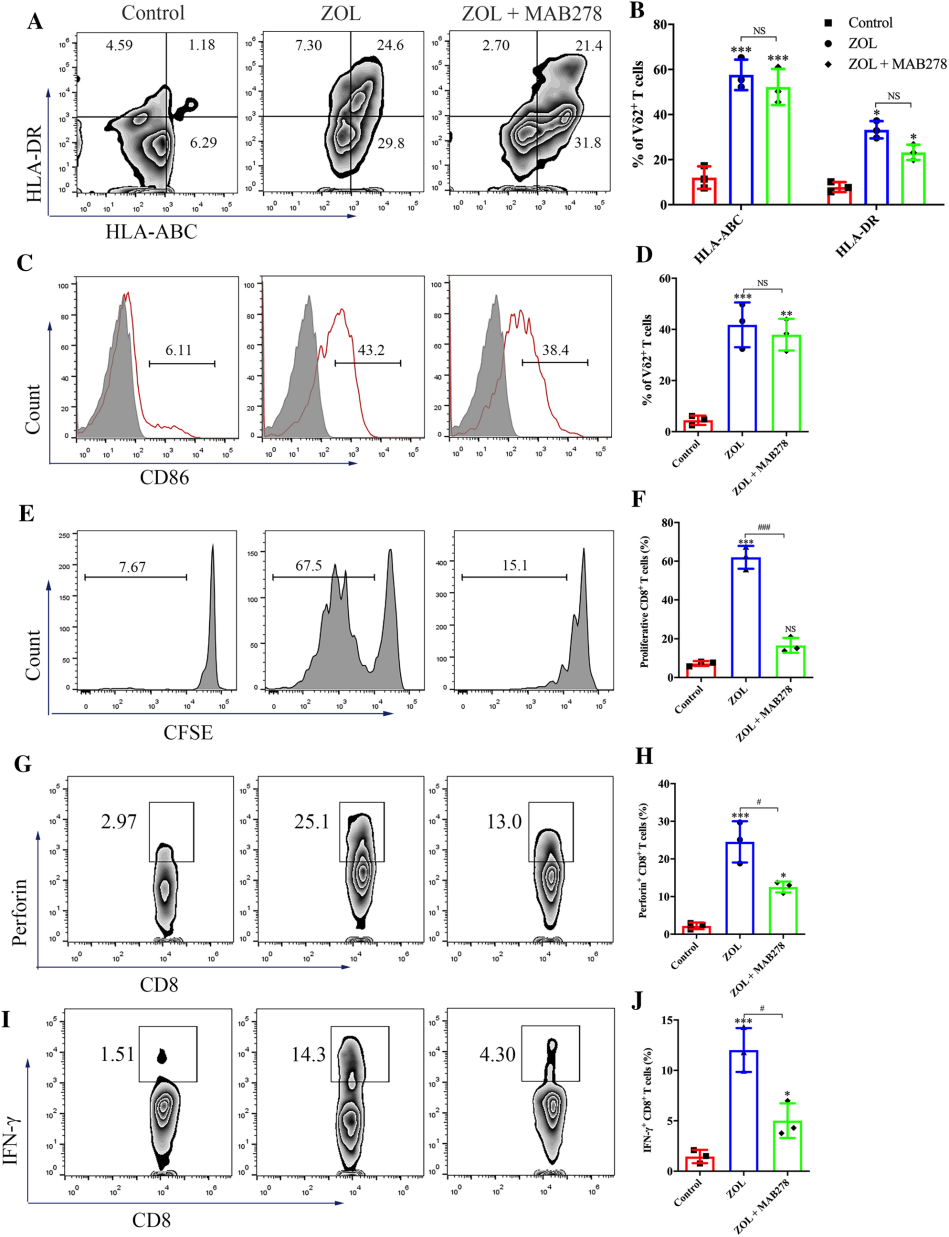
CCL5 promotes the antigen presentation function of γδ T cells. **A**–**D** Resting γδ T cells were treated with ZOL or ZOL plus CCL5 blocking antibody MAB278 (ZOL + MAB278) for 3 days. **A**, B) The expression levels of MHC molecules on γδ T cells were measured using flow cytometry. **C**, **D** The expression levels of CD86 on γδ T cells were measured using flow cytometry. **E**, **F** Resting γδ T cells were treated with ZOL or ZOL + MAB278 for 7 days before MAGEA3 incubation. Then, the γδ T cells were washed and co-cultured with CFSE-labeled CD8^+^ T cells for 10 days at an APC/responder cell ratio of 1:10. Proliferating CFSE^−^ CD8^+^ T cells were measured by flow cytometry. **G**–**J** Resting γδ T cells were treated with ZOL or ZOL + MAB278 for 7 days before MAGEA3 incubation. Then, the γδ T cells were washed and co-cultured with CD8^+^ T cells for 7 days at an APC/responder cell ratio of 1:10. After that, the cells in each group received the same stimulation as the previous one for 4 h followed by further analysis. **G**, **H** Perforin expression levels in CD8^+^ T cells were measured by flow cytometry. **I**, **J** IFN-γ expression levels in CD8^+^ T cells were measured by flow cytometry. Representative data were shown from 2 to 3 independent experiments. All the values were presented as mean ± SD. *p < 0.05, **p < 0.01, ***p < 0.001 versus Control, #*p* < 0.05, ###*p* < 0.001. NS, not significant

Finally, we determined the involvement of JNK activation in ZOL-induced acquisition of antigen-presenting function by γδ T cells. The JNK/MAPK pathway is involved in triggering the release of cytokines and chemokines from immune cells that are essential for antigen presentation, such as CCL5 and IL-1β [30, 31]. As shown in Figure S9A and S9B, in addition to the indicated factors that have been demonstrated to be upregulated in activated γδ T cells [32], the chemokine CCL5, which mediates T cell homing, was found to be highly expressed in ZOL-stimulated γδ T cells. In addition, *CCL5* expression in activated γδ T cells was significantly inhibited by SP600125, indicating the regulatory effect of the JNK pathway on CCL5 generation (Figure S9C). To assess whether the increase in APC-related markers is dependent on CCL5, we used the CCL5 blocking antibody MAB278 and detected little alteration in MHC molecules and CD86 on ZOL-activated γδ T cells (Fig. 7A–D). However, γδ T cells treated with ZOL combined with MAB278 exerted a significantly weaker ability to induce proliferation and perforin and IFN-γ production in CD8^+^ T cells (Fig. 7E–J). Thus, these findings suggest that the JNK activation-mediated antigen-presenting ability of γδ T cells is partially dependent on CCL5.

## Discussion

The key factor determining the efficacy of immunotherapies, including adoptive cell therapy and cancer vaccines, is the generation of cell-mediated immunity, which depends on antigen cross-presentation by APCs and the optimal priming of antigen-specific CD8^+^ T cells [33, 34]. DCs are currently considered to be the most potent APCs in inducing T cell responses and have been widely used in experimental studies [35]. However, DCs are difficult to isolate from peripheral blood in sufficient amount to produce large-scale T cell activation and expansion in vitro, and many of the cytokines and molecules for DC maturation are not available for clinical use. Multiple clinical trials have demonstrated that DC-based antitumor therapies have limited efficacy due to insufficient antigen presentation [36]. In addition, the process of obtaining therapeutic autologous DCs is laborious and costly. These limitations have impeded the widespread use of DC-based therapeutic strategies.

Constant research have been conducted into various DC alternatives that could be essential in inducing favorable immune response. Lymphoblastoid cell lines (LCLs) have been investigated as promising alternative APCs for eliciting ﻿cytotoxic T cell responses against viruses and cancers [37–39]. Most importantly, LCLs can be easily obtained from Epstein-Barr virus (EBV)-positive humans [40]. However, the expression of endogenous EBV antigens interferes with the expansion of antigen-specific T cells that recognize subdominant tumor antigens. Neoantigen-loaded LCLs can stimulate not only neoantigen-specific T cells but also nonspecific T cells [41]. Therefore, high-passage LCLs appear to be unsuitable to serve as APCs due to random non-silent mutations, particularly for the presentation of neoantigens with low immunogenicity [42]. In addition, T cell blasts were found to act as APCs (T-APCs) and have been used to expand antigen-specific CTLs both in vitro and in vivo [43, 44]. T-APCs possess several advantages, including rapid expansion and the ability to be genetically modified [45]. However, T-APCs have been shown to induce T cell tolerance after antigen presentation through various mechanisms [46, 47]. Therefore, there remains the need for a novel and practical source of APCs.

γδ T cells are considered a promising alternative to APCs, independent of their potent innate effector properties [12, 13]. Studies have shown that human γδ T cells were found to be more efficient than DCs in inducing CD4^+^ and CD8^+^ T cell responses [13, 14]. In the present study, γδ T cells, after being stimulated by ZOL, were capable of cross-priming CD8^+^ T cells and inducing significant CD8^+^ T-cell cytotoxicity against osteosarcoma cells. Unlike DCs which posed a major challenge in terms of generating a sufficient number of APCs, a large amount of γδ T cells could be easily acquired in a stable and low-cost manner. In addition, previous clinical trials have demonstrated both the safety and efficacy of γδ T cell-based immunotherapy in allogeneic settings [48], as opposed to DCs which have only been used in autologous settings in the majority of cancer trials to date [49]. As a result, multiple factors contribute to the decreased effectiveness of DC-based immunotherapy in clinical practice and prevent them being widely used. Altogether, the ease of manipulation, excellent cross-presentation ability and feasibility of application in an allogeneic setting make γδ T-APCs a promising alternative APC for cancer immunotherapy.

**Fig. 8:**
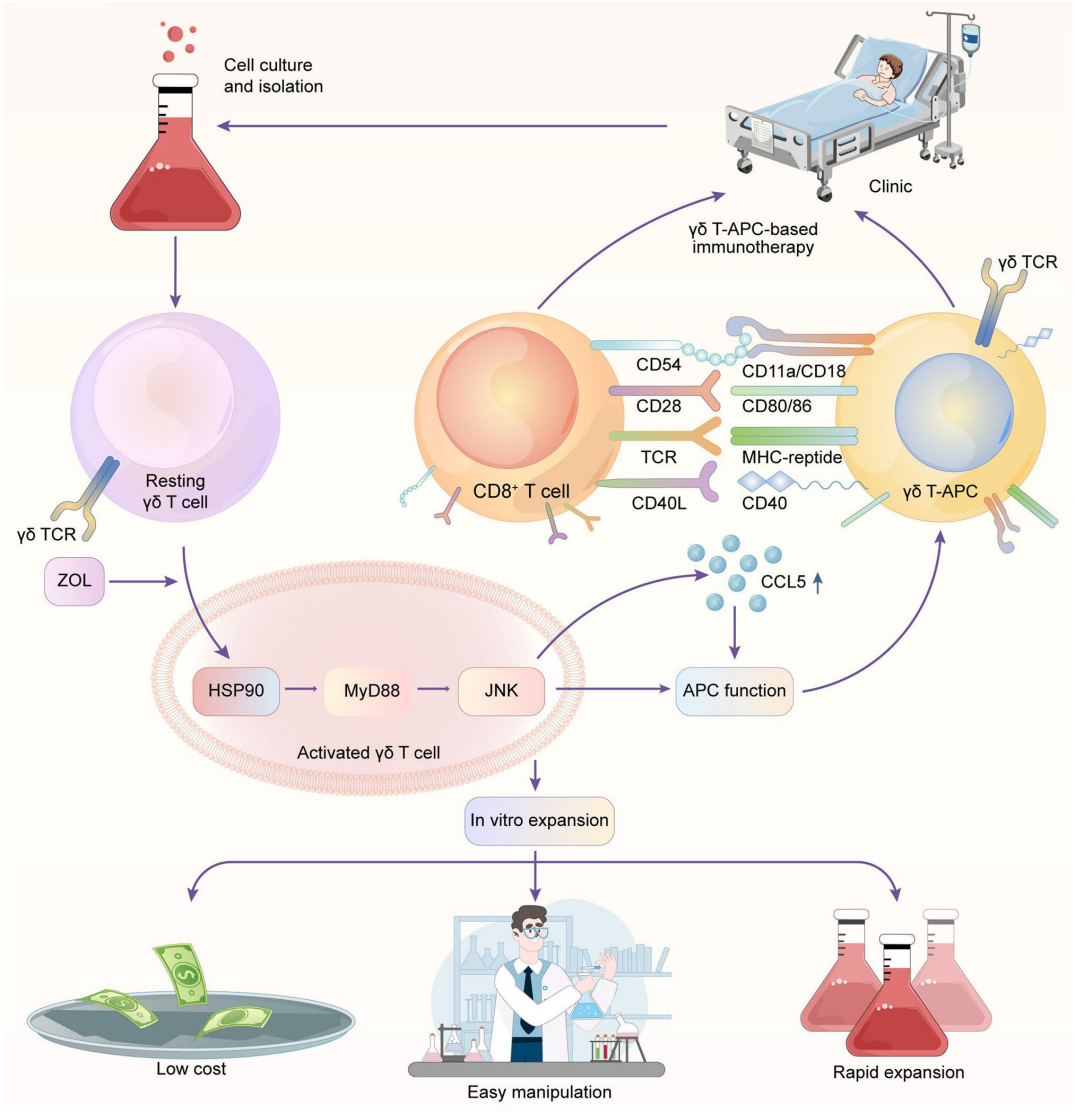
Schematic representation of the effect and mechanism of γδ T cell-mediated antigen presentation. Resting γδ T cells are isolated from PBMCs that are obtained from patients. ZOL stimulation promotes the production of HSP90 in γδ T cells. Then, HSP90 induces the upregulation of MyD88 and subsequent activation of JNK signaling. The HSP90-MyD88-JNK axis would promote the antigen-presenting function of γδ T cells by upregulating APC-related markers and inducing CCL5 expression. With the robust antigen-presenting function and the key advantages, γδ T-APCs hold promising potential as a novel and practical source of APCs for immunotherapy against cancers

Although the unique features of γδ T cells render them an ideal alternative type of APCs, the mechanisms by which γδ T cells develop antigen-presenting phenotypic characteristics remain unclear. In this study, we revealed that HSP90 played a central role in the initiation of the antigen-presenting ability of resting γδ T cells. We verified that HSP90 induced the upregulation of MyD88 and the subsequent activation of JNK signaling. The use of MyD88 and JNK inhibitors completely eliminated the antigen-presenting ability of resting γδ T cells, suggesting that both MyD88 and JNK are necessary for this process. Moreover, JNK-induced CCL5 was shown to be partially responsible for the antigen-presenting ability of γδ T cells, which was probably due to the failed regulation of MHC and co-stimulatory molecules by CCL5. Therefore, we conclude that the HSP90-MyD88-JNK axis induces the antigen-presenting ability of γδ T cells in two ways (Fig. 8). On the one hand, the HSP90-MyD88-JNK axis upregulates MHC and co-stimulatory molecules; on the other hand, the HSP-MyD88-JNK axis induces CCL5 secretion. However, we did not elucidate how CCL5 was involved in the regulation of the antigen-presenting ability of γδ T cells in this study. Future studies are needed to clarify the effect and mechanism of CCL5 in γδ T cell-mediated antigen presentation. Overall, our findings point to the promising perspective of γδ T-APC-based immunotherapy for cancers (Fig. 8).

γδ T cells are increasingly being recognized as key players in cancer immunotherapy, based on recent discoveries related to their powerful therapeutic potential in cancer [50]. Previous studies have demonstrated the exceptional potential of γδ T cells to bridge innate and adaptive immunity. Both our previous and current researches have revealed that γδ T cells play a dual role in inducing direct and indirect antitumor responses. Additionally, γδ T cell infusion has been proved to be safe and well tolerated [51], further indicating the potential of these cells for clinical application.

Despite the extraordinary dual antitumor effects observed, low level of infiltrating γδ T cells in the osteosarcoma specimens was detected (data not shown). To realize the potential of γδ T-APC-based immunotherapy, further studies are needed to promote the homing of γδ T cells by increasing the expression of chemokine receptors or exploring the available technology to achieve targeted delivery of γδ T cells to the tumor site. Once infiltrated into the tumors, activated γδ T cells would lyse target cells upon direct recognition. The released antigens are subsequently taken up by γδ T-APCs and presented to αβ T cells, triggering specific antitumor immune responses. Another promising strategy for γδ T-APC-based immunotherapy is cancer vaccines. γδ T-APCs can be pretreated with defined tumor antigens or tumor extracts from the tumor cells from patients before infusion, depending on the therapeutic regimens that need to be defined during clinical trials. These personalized γδ T-APC-based vaccines would most likely strengthen tumor immunosurveillance by boosting the host immune system and mobilizing tumor-specific T cell responses. In view of so many advantages, we believe that γδ T cells hold high potential as novel APCs for immunotherapy against malignancies.

### Supplementary Information

Below is the link to the electronic supplementary material.

Figure S1. Gene expression profile analysis in resting and activated γδ T cells. (A) Representative flow cytometry showed the percentage of Vδ2 T cells in human PBMCs before and after purification. (B) Heatmap of gene expression profile by RNA-seq showed differentially expressed mRNAs in activated γδ T cells, as compared with resting γδ T cells. (C) Gene ontology annotation of the target genes for those differentially expressed mRNAs was shown (JPG 553 KB)

Figure S2. Zoledronate-activated γδ T cells upregulate APC-related molecules. (A, B) Resting γδ T cells isolated from PBMCs were cultured for 7 days in the presence or absence of zoledronate. Expressions of MHC, co-stimulatory and adhesion molecules in resting and activated γδ T cells were measured by flow cytometry. All the values were presented as mean ± SD. ***p < 0.001 vs. resting γδ T cells. (C) Time courses of the expression of MHC and co-stimulatory molecules on γδ T cells after zoledronate stimulation. All the values were presented as mean ± SD. **p < 0.01, ***p < 0.001, #p < 0.05, ##p < 0.01, ###p < 0.001, vs. Day 0 of the corresponding group. (D) Expression and distribution of HLA-ABC (red) in γδ T cells treated with zoledronate for indicated time were detected by immunofluorescence. Mature DCs were set as the positive control. The nuclei were counterstained with DAPI (blue). Scale bar: 2 µm. (E) Ex vivo relative proliferation rate of γδ T cells and DCs were determined by cell counting at indicated time after activation (JPG 1545 KB)

Figure S3. Subcellular transport of antigen in γδ T-APCs. γδ T-APCs were incubated with FITC-conjugated MAGEA3 long peptide for 1 h and then washed extensively to remove unbound peptide. The cells were cultured for an additional 0 h, 1 h, 3 h, 6 h or 24 h before fixation followed by immunostaining with antibodies recognizing specific organelles (EEA1 for early endosome, Rab7 for late endosome, KDEL for endoplasmic reticulum and LAMP1 for the lysosome) (JPG 922 KB)

Figure S4. γδ T-APCs pulsed with HER2 or lysate induce the cytotoxicity of CD8+ T cells against osteosarcoma cell lines or primary osteosarcoma cells. γδ T cells stimulated by zoledronate and IL-2 for 10 days were used as γδ T-APCs. (A) γδ T-APCs or DCs were, respectively, pretreated for 2 h with 10 μg/ml HER2 peptide (γδ T-HER2 and DC-HER2) or irrelevant peptide (γδ T-IP) and then washed extensively before co-culture. CD8+ T cells isolated from PBMCs were, respectively, co-cultured with peptide non-pulsed γδ T cells, γδ T-IP, γδ T-HER2 or DC-HER2 for 14 days at an APC/responder cell ratio of 1:10. Then, T cells were harvested and co-cultured with HER2-transfected or non-transfected (NT) osteosarcoma cell lines for 4 h at an effector/target cell ratio of 10:1. The antitumor effect was measured by LDH assay. (B) γδ T-APCs were pulsed with tumor lysates (γδ T-Lysate) from osteosarcoma cell lines or primary tumor cells from osteosarcoma patients and then washed extensively before being co-cultured with CD8+ T cells for 14 days at an APC/responder cell ratio of 1:10. Then, the CD8+ T cells were harvested and co-cultured with osteosarcoma cell lines or primary tumor cells for 4 h at an effector/target cell ratio of 10:1. The antitumor effect was measured by LDH assay. All the values were presented as mean ± SD. **p < 0.01, ***p < 0.001 vs. Control. NS, not significant (JPG 731 KB)

Figure S5. Phenotypes of HOS cells after transfection. (A) Western blot analysis showed the expression levels of MAGEA3 in non-transfected (NT) and transfected cells. (B) Non-transfected cells or transfected cells were inoculated subcutaneously into the left thighs of NOD-SCID mice. After 10 days, mice were imaged with the in vivo imaging system to determine the transfection of luciferase (JPG 126 KB)

Figure S6. HSP90 inhibitor has little effect in the proliferation or apoptosis of γδ T cells. (A, B) Resting γδ T cells were treated with zoledronate (ZOL) or ZOL plus HSP90 inhibitor luminespib (ZOL+LUM) for indicated days. (A) The proportion of γδ T cells was measured by flow cytometry. (B) The proportion of live γδ T cells was measured by flow cytometry using 7-AAD staining. All the values were presented as mean ± SD (JPG 121 KB)

Figure S7. TLR4 agonist upregulates APC-related phenotypes in γδ T cells. (A-D) Resting γδ T cells were treated with TLR4 and TLR3 agonists LPS and PIC, respectively, for 3 days. (A, B) The expression levels of MHC molecules on γδ T cells were measured using flow cytometry. (C, D) The expression levels of CD86 on γδ T cells were measured using flow cytometry. All the values were presented as mean ± SD. ***p < 0.001 vs. Control. NS, not significant vs. Control (JPG 141 KB)

Figure S8. Mechanism of γδ T cell-mediated antigen presentation. (A) Resting γδ T cells were cultured in the absence of zoledronate (ZOL) for indicated days. The expression levels of HSP90α, HSP90β, GRP94 and TRAP1 were detected by western blot. (B) KEGG pathway analysis of the DEGs in resting and ZOL-activated γδ T cells. (C) Resting γδ T cells were treated with 1 μM ZOL for indicated days. The expression levels of p-P38, P38, p-ERK and ERK were detected by western blot. (D-G) Resting γδ T cells were treated with ZOL, ZOL plus P38 inhibitor SB203580 or ZOL plus ERK inhibitor PD98059 for 3 days. (D, E) The expression levels of MHC molecules on γδ T cells were measured using flow cytometry. (F, G) The expression levels of CD86 on γδ T cells were measured using flow cytometry. All the values were presented as mean ± SD. ***p < 0.001 vs. Control. NS, not significant (JPG 510 KB)

Figure S9. Zoledronate (ZOL) stimulation promotes CCL5 production of γδ T cells. (A) RNA-seq analysis showed the DEGs of cytokines and chemokines in ZOL-stimulated γδ T cells. (B) PCR analysis showed the mRNA levels of cytokines and chemokines in resting and ZOL-stimulated γδ T cells. (C) Resting γδ T cells were treated with ZOL or ZOL + SP for 7 days. The mRNA level of CCL5 was measured by RT-PCR. **p < 0.01, ***p < 0.001 vs. Control. ##p < 0.01. NS, not significant. SP, JNK inhibitor SP600125 (JPG 150 KB)

Supplementary file10 (DOCX 14 KB)

Supplementary file11 (DOCX 15 KB)

## Data Availability

All data supporting this study are presented within the paper and/or Supplementary Materials. The original datasets are available from the corresponding author upon request.
