# Supplementary figures and images for: Human γδ T cells induce CD8+ T cell antitumor responses via antigen-presenting effect through HSP90-MyD88-mediated activation of JNK
Source: Cancer Immunol Immunother. 2023 Jan 21;72(6):1803–21. doi: 10.1007/s00262-023-03375-w (PMC10198898; doi:10.1007/s00262-023-03375-w)

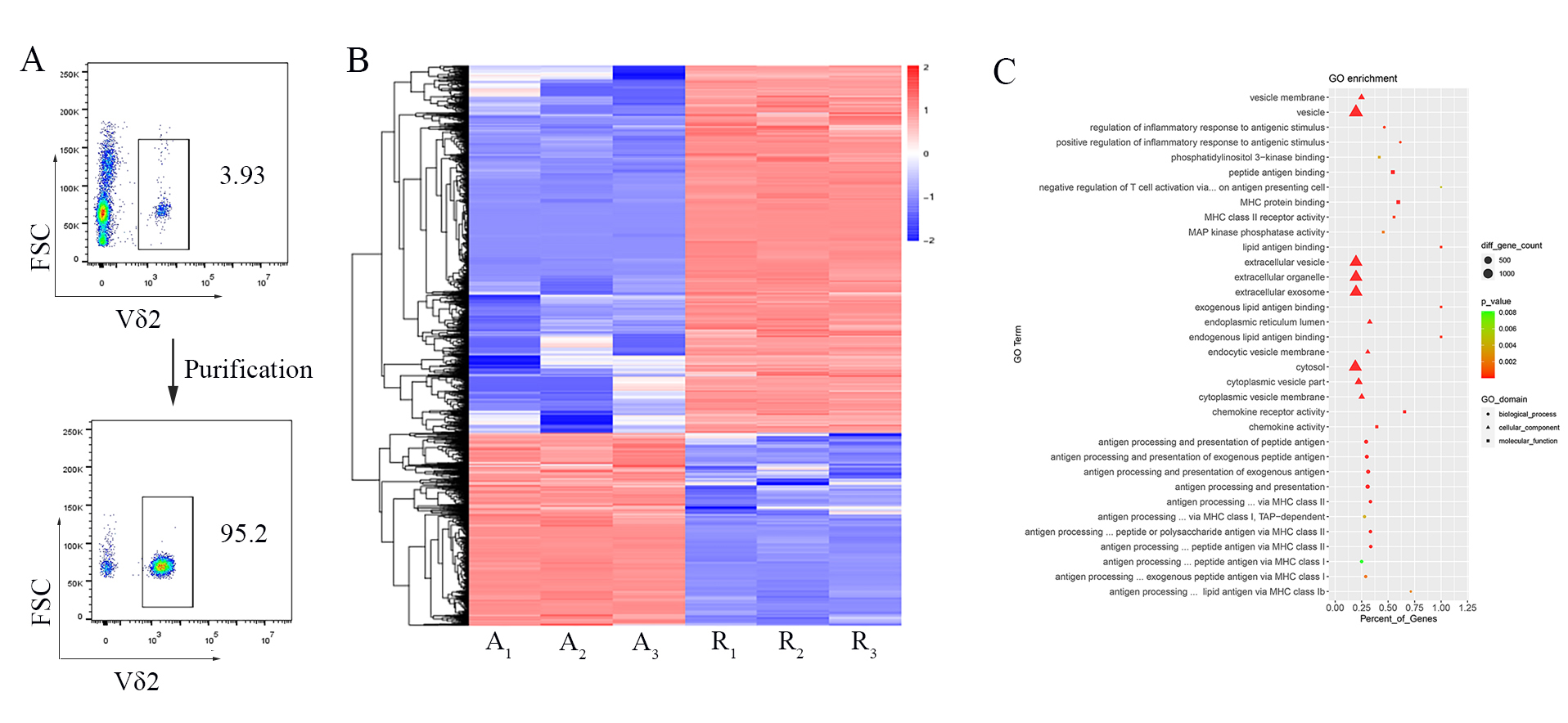

Supplement: Supplementary file 1 — Figure S1. Gene expression profile analysis in resting and activated γδ T cells. (A) Representative flow cytometry showed the percentage of Vδ2 T cells in human PBMCs before and after purification. (B) Heatmap of gene expression profile by RNA-seq showed differentially expressed mRNAs in activated γδ T cells, as compared with resting γδ T cells. (C) Gene ontology annotation of the target genes for those differentially expressed mRNAs was shown (JPG 553 KB) [file 262_2023_3375_MOESM1_ESM.jpg]

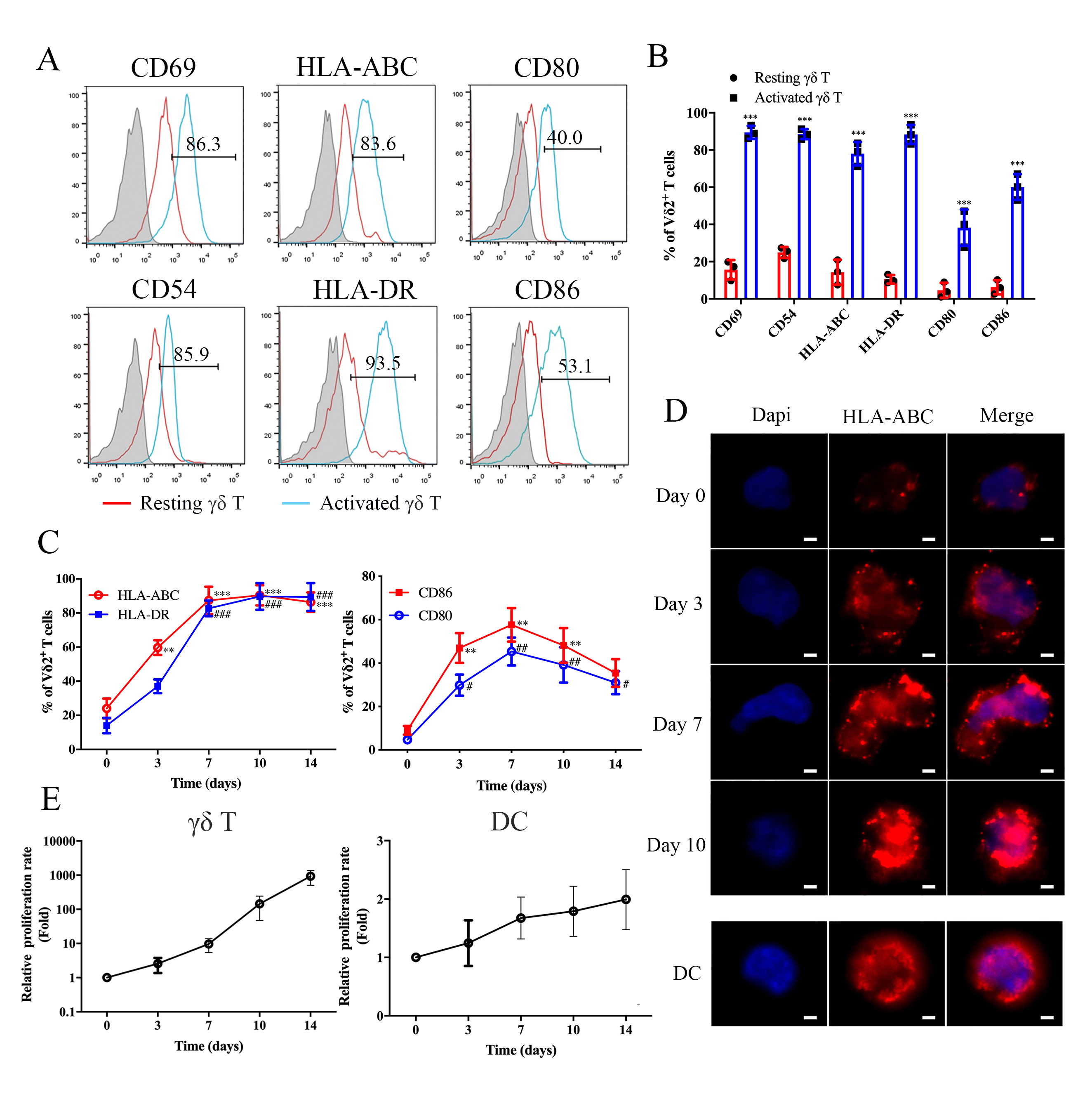

Supplement: Supplementary file 2 — Figure S2. Zoledronate-activated γδ T cells upregulate APC-related molecules. (A, B) Resting γδ T cells isolated from PBMCs were cultured for 7 days in the presence or absence of zoledronate. Expressions of MHC, co-stimulatory and adhesion molecules in resting and activated γδ T cells were measured by flow cytometry. All the values were presented as mean ± SD. ***p < 0.001 vs. resting γδ T cells. (C) Time courses of the expression of MHC and co-stimulatory molecules on γδ T cells after zoledronate stimulation. All the values were presented as mean ± SD. **p < 0.01, ***p < 0.001, #p < 0.05, ##p < 0.01, ###p < 0.001, vs. Day 0 of the corresponding group. (D) Expression and distribution of HLA-ABC (red) in γδ T cells treated with zoledronate for indicated time were detected by immunofluorescence. Mature DCs were set as the positive control. The nuclei were counterstained with DAPI (blue). Scale bar: 2 µm. (E) Ex vivo relative proliferation rate of γδ T cells and DCs were determined by cell counting at indicated time after activation (JPG 1545 KB) [file 262_2023_3375_MOESM2_ESM.jpg]

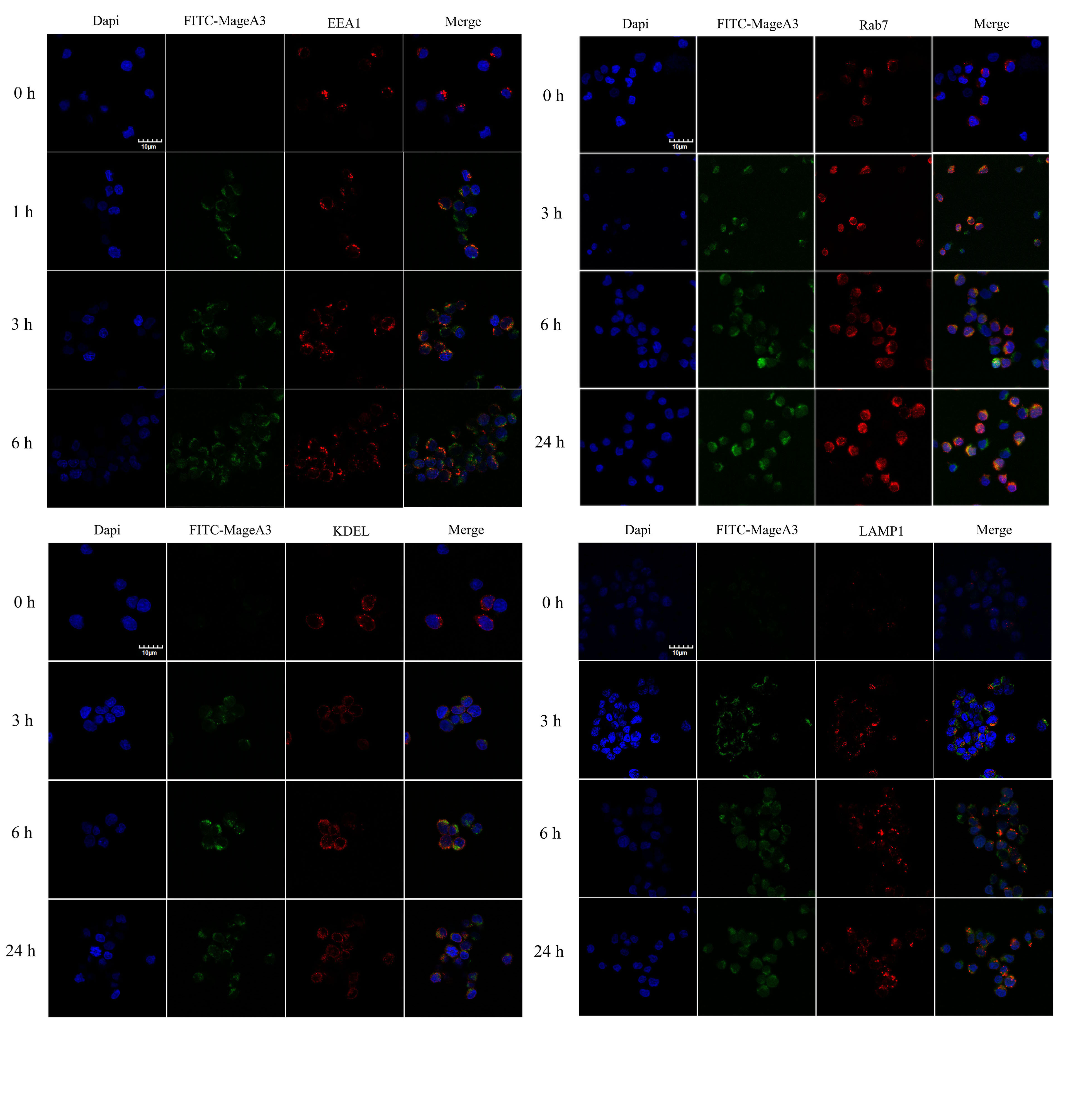

Supplement: Supplementary file 3 — Figure S3. Subcellular transport of antigen in γδ T-APCs. γδ T-APCs were incubated with FITC-conjugated MAGEA3 long peptide for 1 h and then washed extensively to remove unbound peptide. The cells were cultured for an additional 0 h, 1 h, 3 h, 6 h or 24 h before fixation followed by immunostaining with antibodies recognizing specific organelles (EEA1 for early endosome, Rab7 for late endosome, KDEL for endoplasmic reticulum and LAMP1 for the lysosome) (JPG 922 KB) [file 262_2023_3375_MOESM3_ESM.jpg]

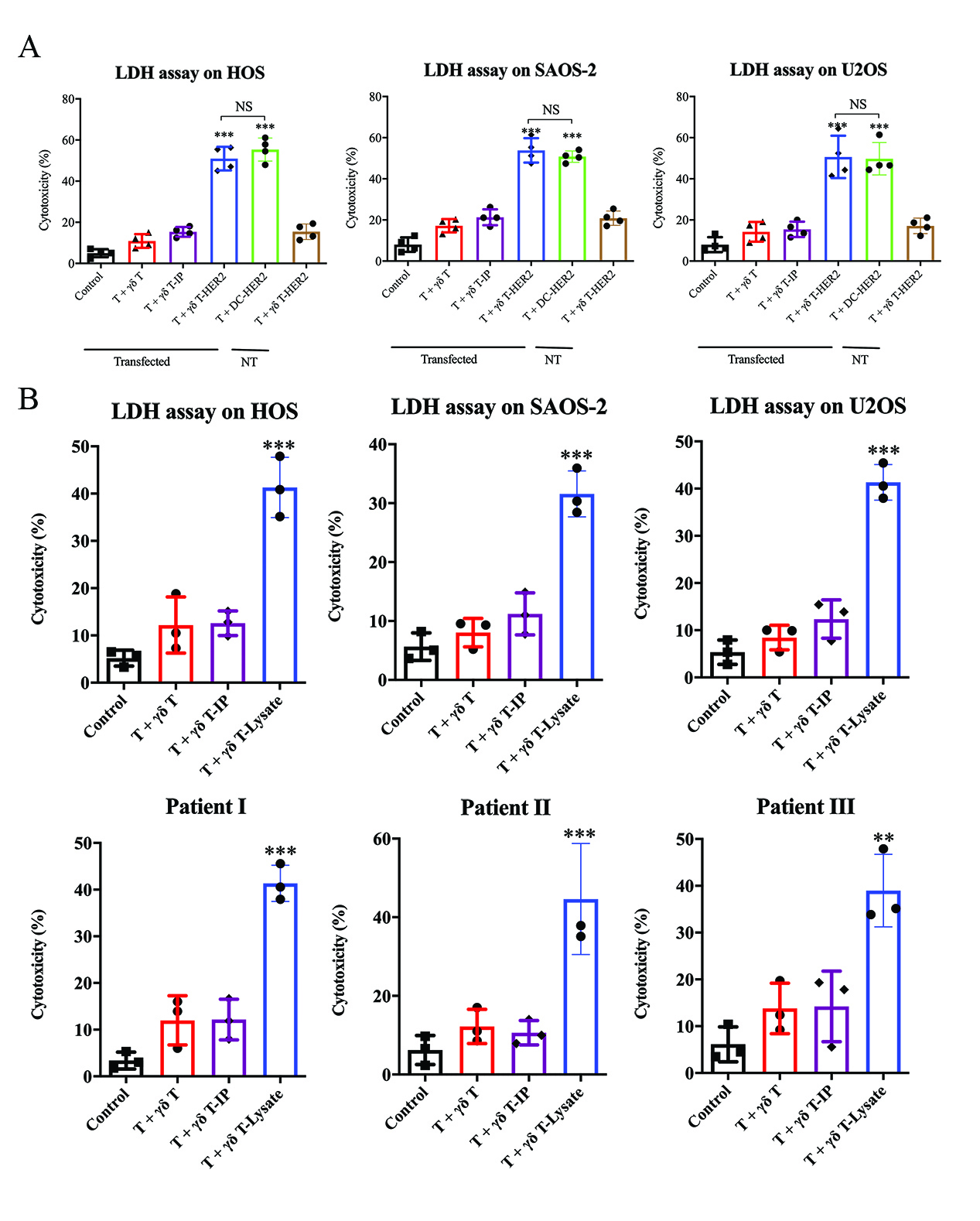

Supplement: Supplementary file 4 — Figure S4. γδ T-APCs pulsed with HER2 or lysate induce the cytotoxicity of CD8+ T cells against osteosarcoma cell lines or primary osteosarcoma cells. γδ T cells stimulated by zoledronate and IL-2 for 10 days were used as γδ T-APCs. (A) γδ T-APCs or DCs were, respectively, pretreated for 2 h with 10 μg/ml HER2 peptide (γδ T-HER2 and DC-HER2) or irrelevant peptide (γδ T-IP) and then washed extensively before co-culture. CD8+ T cells isolated from PBMCs were, respectively, co-cultured with peptide non-pulsed γδ T cells, γδ T-IP, γδ T-HER2 or DC-HER2 for 14 days at an APC/responder cell ratio of 1:10. Then, T cells were harvested and co-cultured with HER2-transfected or non-transfected (NT) osteosarcoma cell lines for 4 h at an effector/target cell ratio of 10:1. The antitumor effect was measured by LDH assay. (B) γδ T-APCs were pulsed with tumor lysates (γδ T-Lysate) from osteosarcoma cell lines or primary tumor cells from osteosarcoma patients and then washed extensively before being co-cultured with CD8+ T cells for 14 days at an APC/responder cell ratio of 1:10. Then, the CD8+ T cells were harvested and co-cultured with osteosarcoma cell lines or primary tumor cells for 4 h at an effector/target cell ratio of 10:1. The antitumor effect was measured by LDH assay. All the values were presented as mean ± SD. **p < 0.01, ***p < 0.001 vs. Control. NS, not significant (JPG 731 KB) [file 262_2023_3375_MOESM4_ESM.jpg]

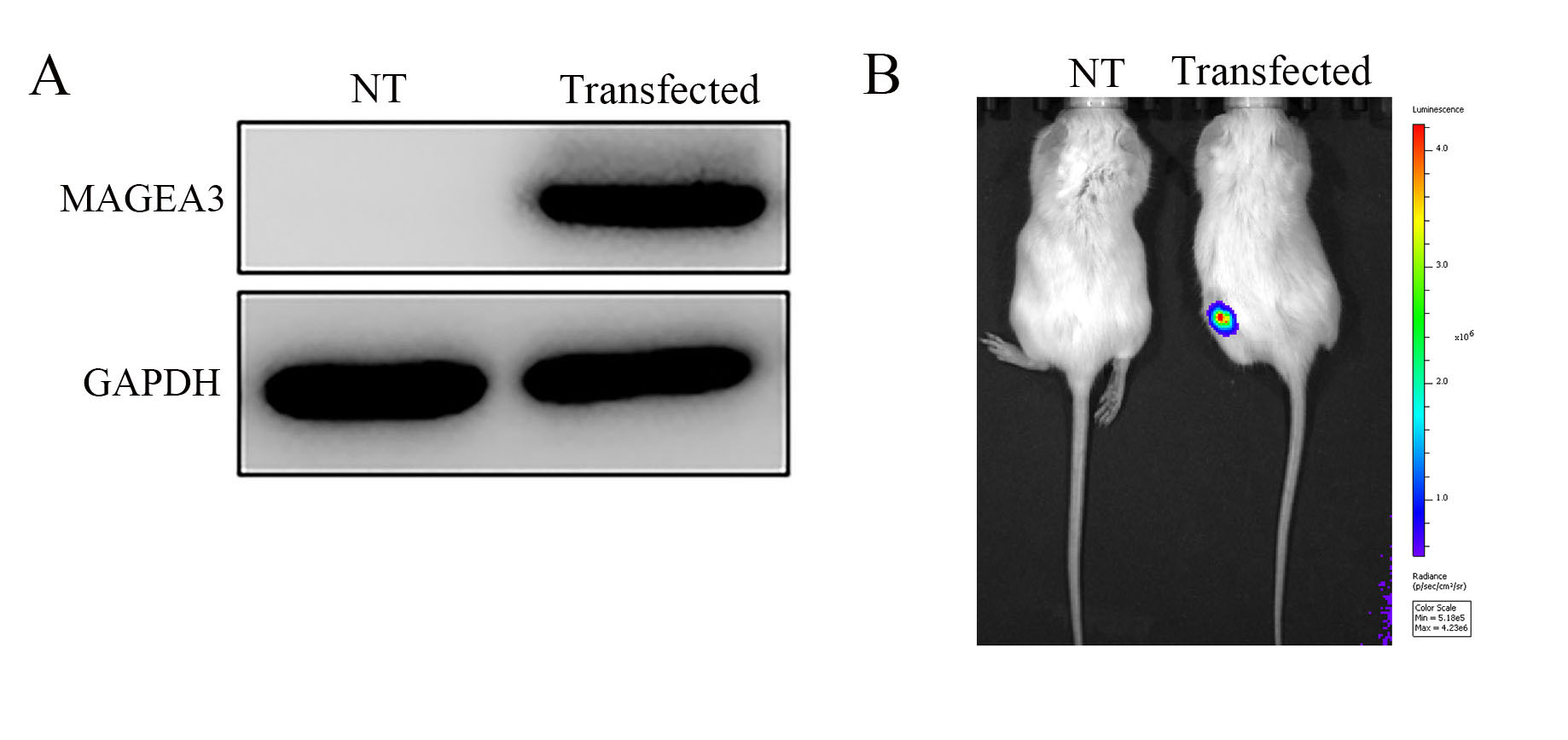

Supplement: Supplementary file 5 — Figure S5. Phenotypes of HOS cells after transfection. (A) Western blot analysis showed the expression levels of MAGEA3 in non-transfected (NT) and transfected cells. (B) Non-transfected cells or transfected cells were inoculated subcutaneously into the left thighs of NOD-SCID mice. After 10 days, mice were imaged with the in vivo imaging system to determine the transfection of luciferase (JPG 126 KB) [file 262_2023_3375_MOESM5_ESM.jpg]

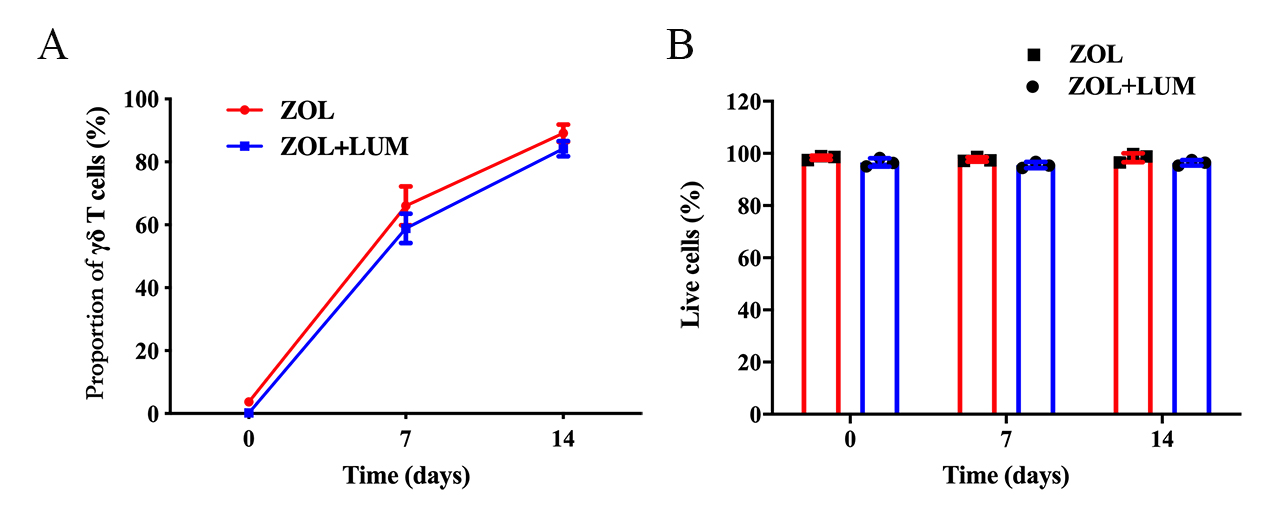

Supplement: Supplementary file 6 — Figure S6. HSP90 inhibitor has little effect in the proliferation or apoptosis of γδ T cells. (A, B) Resting γδ T cells were treated with zoledronate (ZOL) or ZOL plus HSP90 inhibitor luminespib (ZOL+LUM) for indicated days. (A) The proportion of γδ T cells was measured by flow cytometry. (B) The proportion of live γδ T cells was measured by flow cytometry using 7-AAD staining. All the values were presented as mean ± SD (JPG 121 KB) [file 262_2023_3375_MOESM6_ESM.jpg]

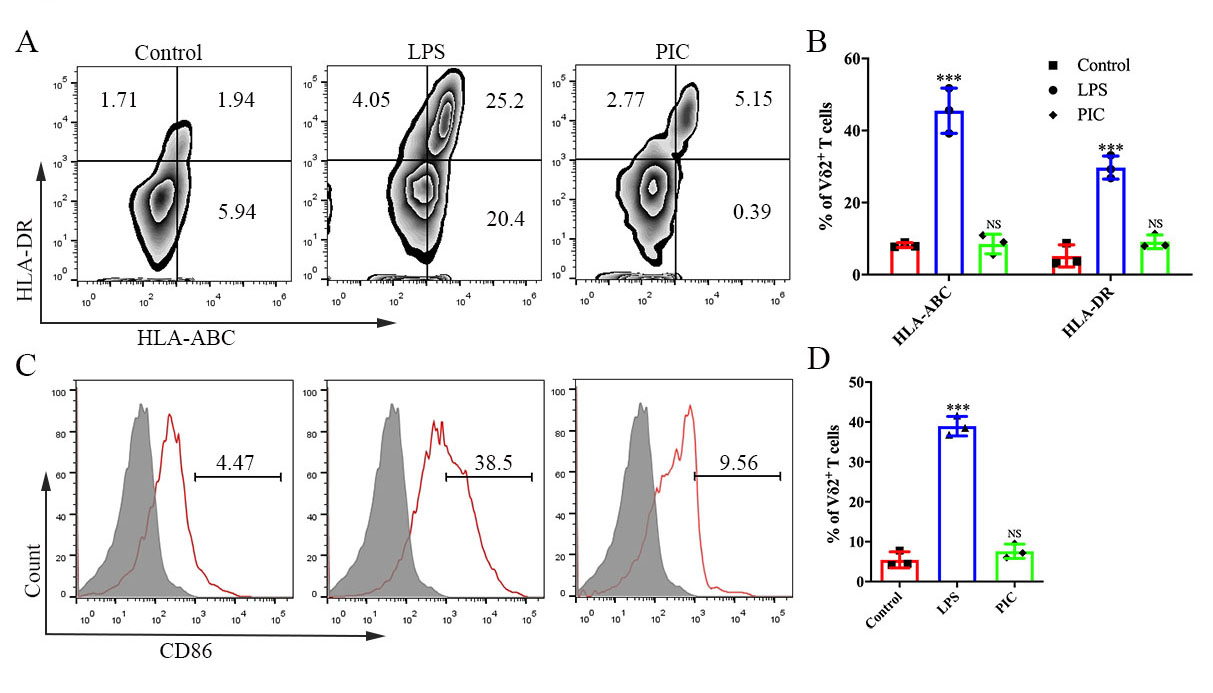

Supplement: Supplementary file 7 — Figure S7. TLR4 agonist upregulates APC-related phenotypes in γδ T cells. (A-D) Resting γδ T cells were treated with TLR4 and TLR3 agonists LPS and PIC, respectively, for 3 days. (A, B) The expression levels of MHC molecules on γδ T cells were measured using flow cytometry. (C, D) The expression levels of CD86 on γδ T cells were measured using flow cytometry. All the values were presented as mean ± SD. ***p < 0.001 vs. Control. NS, not significant vs. Control (JPG 141 KB) [file 262_2023_3375_MOESM7_ESM.jpg]

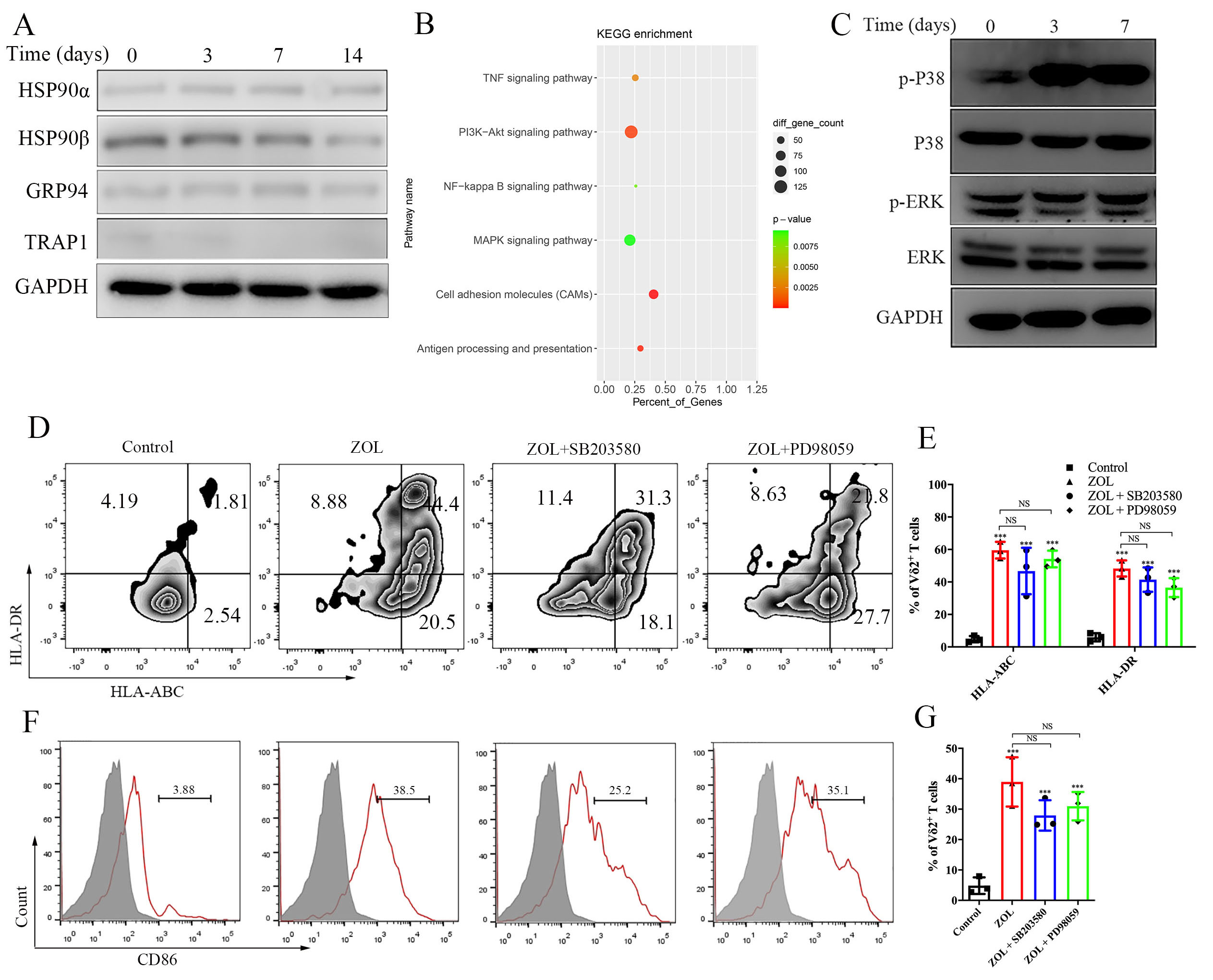

Supplement: Supplementary file 8 — Figure S8. Mechanism of γδ T cell-mediated antigen presentation. (A) Resting γδ T cells were cultured in the absence of zoledronate (ZOL) for indicated days. The expression levels of HSP90α, HSP90β, GRP94 and TRAP1 were detected by western blot. (B) KEGG pathway analysis of the DEGs in resting and ZOL-activated γδ T cells. (C) Resting γδ T cells were treated with 1 μM ZOL for indicated days. The expression levels of p-P38, P38, p-ERK and ERK were detected by western blot. (D-G) Resting γδ T cells were treated with ZOL, ZOL plus P38 inhibitor SB203580 or ZOL plus ERK inhibitor PD98059 for 3 days. (D, E) The expression levels of MHC molecules on γδ T cells were measured using flow cytometry. (F, G) The expression levels of CD86 on γδ T cells were measured using flow cytometry. All the values were presented as mean ± SD. ***p < 0.001 vs. Control. NS, not significant (JPG 510 KB) [file 262_2023_3375_MOESM8_ESM.jpg]

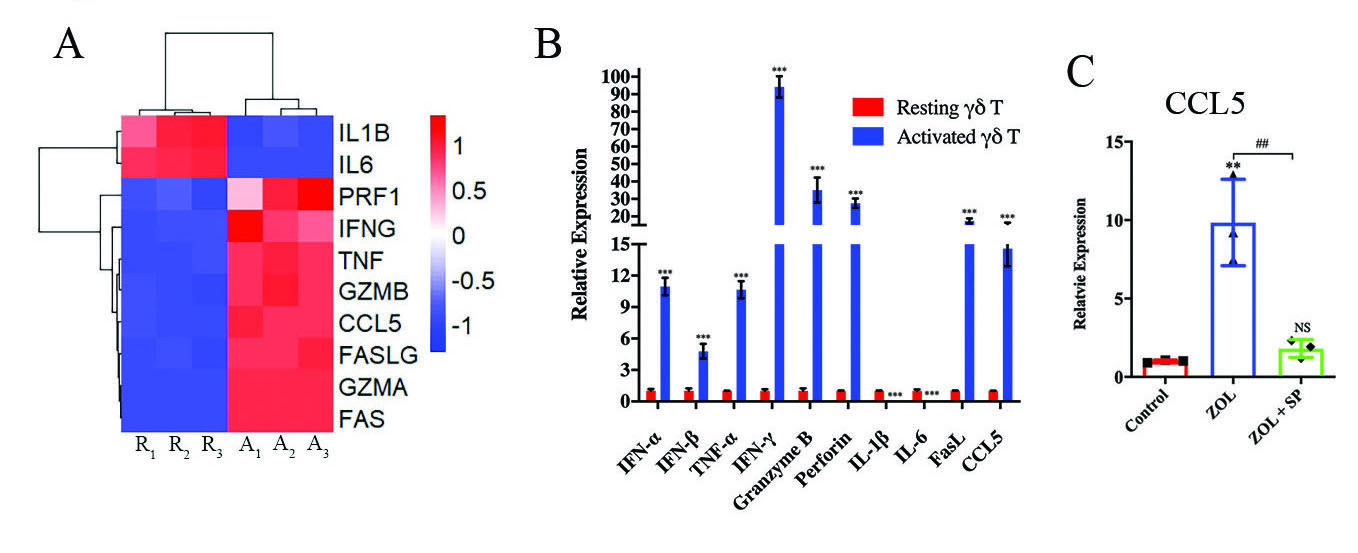

Supplement: Supplementary file 9 — Figure S9. Zoledronate (ZOL) stimulation promotes CCL5 production of γδ T cells. (A) RNA-seq analysis showed the DEGs of cytokines and chemokines in ZOL-stimulated γδ T cells. (B) PCR analysis showed the mRNA levels of cytokines and chemokines in resting and ZOL-stimulated γδ T cells. (C) Resting γδ T cells were treated with ZOL or ZOL + SP for 7 days. The mRNA level of CCL5 was measured by RT-PCR. **p < 0.01, ***p < 0.001 vs. Control. ##p < 0.01. NS, not significant. SP, JNK inhibitor SP600125 (JPG 150 KB) [file 262_2023_3375_MOESM9_ESM.jpg]
