# Supplementary material for: Human γδ T cells induce CD8+ T cell antitumor responses via antigen-presenting effect through HSP90-MyD88-mediated activation of JNK
Source: Cancer Immunol Immunother. 2023 Jan 21;72(6):1803–21. doi: 10.1007/s00262-023-03375-w (PMC10198898; doi:10.1007/s00262-023-03375-w)
Supplement: Supplementary file 10 — Supplementary file10 (DOCX 14 KB) [file 262_2023_3375_MOESM10_ESM.docx]

| Primer | Forward | Reverse |
| --- | --- | --- |
| *HSP90α* | TTGGTTACTTCCCCGTGCTG | GCCTTTTGCCGTAGGGTTTC |
| *HSP90β* | TATCCGAGGTGTGGTGGACT | GCTTCCGTACTCGTTCCACA |
| *HSP70* | AGCCAAGAAGGCAAAAGTGA | CCACTGCGTTCTTAGCATCA |
| *HSP60* | AGGCAAGGGTGAAAAATCCCA | AAGCAATGCACAACCACCAC |
| *HSP40* | TCCCAGACCCTGTACACTCC | TTGCTGGAGTCACTCACTGG |
| *GAPDH* | AGGTCGGTGTGAACGGATTTG | TGTAGACCATGTAGTTGAGGTCA |
| *IFN-α* | GTGAGGAAATACTTCCAAAGAATCAC | TCTCATGATTTCTGCTCTGACAA |
| *IFN-β* | AGCTGAAGCAGTTCCAGAAG | AGTCTCATTCCAGCCAGTGC |
| *TNF-α* | CCCAGGCAGTCAGATCATCTTC | AGCTGCCCCTCAGCTTGA |
| *IFN-γ* | TGGCTTTTCAGCTCTGCATC | CCGCTACATCTGAATGACCTG |
| *Granzyme B* | TGGGGGACCCAGAGATTAAAA | TTTCGTCCATAGGAGACAATGC |
| *Perforin* | CGCCTACCTCAGGCTTATCTC | CCTCGACAGTCAGGCAGTC |
| *IL1-β* | CCACAGACCTTCCAGGAGAATG | GTGCAGTTCAGTGATCGTACAGG |
| *IL10* | GCCTAACATGCTTCGAGATC | CTCATGGCTTTGTAGATGCC |
| *IL-6* | GTACATCCTCGACGGCATCT | GTGCCTCTTTGCTGCTTTCAC |
| *FasL* | TCAATGAAACTGGGCTGTACTTT | AGAGTTCCTCATGTAGACCTTGT |
| *IL2* | GAATCCCAAACTCACCAGGATGCTC | TAGCACTTCCTCCAGAGGTTTGAGT |
| *CCL-5* | GAGTATTTCTACACCAGTGGCAAG | TCCCGAACCCATTTCTTCTCT |

**Table S1 ﻿Sequences of primers for PCR**
