# Supplementary material for: Human γδ T cells induce CD8+ T cell antitumor responses via antigen-presenting effect through HSP90-MyD88-mediated activation of JNK
Source: Cancer Immunol Immunother. 2023 Jan 21;72(6):1803–21. doi: 10.1007/s00262-023-03375-w (PMC10198898; doi:10.1007/s00262-023-03375-w)
Supplement: Supplementary file 11 — Supplementary file11 (DOCX 15 KB) [file 262_2023_3375_MOESM11_ESM.docx]

| **Table S2 Specimen Information** | | | | | |
| --- | --- | --- | --- | --- | --- |
|  |  |  |  |  |  |
|  | |  | | | |
| Name | | Age | Gender | Location | Feature of specimen |
| Patient 1 | | 21 | Male | left tibia | pre-chemotherapy |
| Patient 2 | | 15 | Male | right ilium | pre-chemotherapy |
| Patient 3 | | 13 | Female | right femur | pre-chemotherapy |
